# Urban form and scale shaped the agroecology of early ‘cities’ in northern Mesopotamia, the Aegean and Central Europe

**DOI:** 10.1111/joac.12497

**Published:** 2022-05-31

**Authors:** Amy K. Styring, Chris U. Carmona, Valasia Isaakidou, Angeliki Karathanou, Geoff K. Nicholls, Anaya Sarpaki, Amy Bogaard

**Affiliations:** ^1^ School of Archaeology University of Oxford Oxford UK; ^2^ Department of Statistics University of Oxford Oxford UK; ^3^ Laboratory for Interdisciplinary Archaeological Research (LIRA), Department of Archaeology Aristotle University of Thessaloniki Thessaloniki Greece; ^4^ Independent Researcher Chania Greece

**Keywords:** archaeology, Bayesian, cereal cultivation, nitrogen isotope values, semi‐modular inference

## Abstract

Agricultural extensification refers to an expansive, low‐input production strategy that is land rather than labour limited. Here, we present a robust method, using the archaeological proxies of cereal grain nitrogen isotope values and settlement size, to investigate the relationship between agricultural intensity and population size at Neolithic to Bronze/Iron Age settlement sites in northern Mesopotamia, the Aegean and south‐west Germany. We conclude that urban form—in particular, *density* of occupation—as well as scale shaped the agroecological trajectories of early cities. Whereas high‐density urbanism in northern Mesopotamia and the Aegean entailed radical agricultural extensification, lower density urbanism in south‐west Germany afforded more intensive management of arable land. We relate these differing agricultural trajectories to long‐term urban growth/collapse cycles in northern Mesopotamia and the Aegean, on the one hand, and to the volatility of early Iron Age elite power structures and urban centralization in south‐west Germany, on the other.

## 1 INTRODUCTION

The emergence of cities posed new social and ecological challenges, many of which (e.g., wealth inequality and food security) persist in contemporary highly urbanized societies. Establishing how the unprecedented aggregation of people—including those not involved in food production—were fed has been a key preoccupation of archaeologists for decades (e.g., Childe, 1950; Halstead, 1999; Wilkinson, 1994), with implications for the political economies of early urban societies. The conventional understanding is that urbanization involved agricultural ‘intensification’ (e.g., Renfrew, 1982; Wilkinson, 1994), but this term can take on different meanings. ‘Intensification’ strictly refers to greater labour investment or *labour intensity* per unit area in order to achieve greater yields on a given area of land (e.g., Brookfield, 1972), but it is sometimes used to mean cultivation of larger tracts of land and thus larger scales of production—that is, greater *land intensity* (cf. Stanish, 2006). Disentangling these different agronomic strategies archaeologically offers new insights into the long‐term evolution of agrarian practices and political economies associated with urban forms. This *longue durée* perspective provides a fresh foundation for debates surrounding the intersection of agrarian change and political economy, both historically and today, which feature in the *Journal of Agrarian Change*. Discussion of how the organization of agricultural labour and land shape power relations in rural and urban settings around the globe often raises questions about the nature of pre‐industrial farming (e.g., Hecht, 2007; Larcom, 2017; Paz, 2020). A special issue considering the extent of agrarian and political continuity from the (urbanized) Roman to (variously de‐urbanized) post‐Roman world explicitly highlighted “the need to pay closer attention to means and technical modes of production and their social implications when studying a historical society” (Sarris, 2009, p. 20). The approach we take here responds to precisely this need, while extending the field of enquiry back to the beginnings of urbanism in western Eurasia.

The growing of crops on an expansive scale, facilitated by the use of specialized draught animals, is attested in documentary records of institutional involvement in farming in Bronze Age Mesopotamia and the Aegean (e.g., Halstead, 1995; Postgate, 1992, pp. 115, 149, 189). In such land‐intensive systems, where land is sufficient to permit radical expansion, yields per unit area are low relative to labour‐intensive production, which features thorough tillage, weeding and manuring. However, the greater absolute scale of land‐intensive farming enables production of larger surpluses overall. In a recent study (Bogaard et al., 2019) of western Asia and Europe from the ninth millennium BC to early first millennium AD, it was shown that *labour*‐intensive agroecologies were associated with low material wealth inequalities, whereas *land*‐intensive agricultural strategies were linked with higher inequality levels. This is because land is a monopolizable form of wealth that is readily passed down from one generation to the next, whereas labour‐intensive strategies are labour limited (i.e., production is tied to labour inputs) and the regular provision of (free) labour cannot be guaranteed intergenerationally or even during the lifespan of a household (e.g., Borgerhoff Mulder et al., 2009; Halstead, 2014, pp. 95–304). Moreover, it has been argued that extensive, land‐intensive agriculture went hand‐in‐hand with elites and the emergence of urban, hierarchical societies (Halstead, 1990, 1992, 1994). As well as providing access to specialized plough oxen that could not have been supported by individual households, elite‐managed institutions would also have facilitated access to a supply of landless labour at harvest time, which would otherwise be a bottleneck for surplus production (Halstead, 1995).

Archaeological evidence for unspecialized animal tillage by female cattle (e.g., Halstead & Isaakidou, 2011; Isaakidou, 2006) and intensive manuring (Bogaard et al., 2013; Styring, Maier, et al., 2016; Vaiglova et al., 2020) imply intensive, *labour*‐limited agriculture, whereas specialized draught oxen (e.g., Halstead & Isaakidou, 2011) and low inputs of manure (Styring, Charles, et al., 2017) are compatible with *land*‐limited extensive agriculture. Such proxies thus provide a means of exploring how agrarian practices changed and how they relate to urbanization processes. In this paper, we focus on agricultural practices associated with cereal cultivation, because cereals lend themselves to large scale, state‐controlled production that could have supported increasing urban populations (e.g., Diffey et al., 2020; Scott, 2017).

A study of early urbanism in northern Mesopotamia (Styring, Charles, et al., 2017) found that expanding settlement size, taken as a proxy for population, was associated with a decrease in cereal nitrogen isotope (*δ* ^15^N) values, used as a proxy for manuring inputs per unit area. This study thus provided evidence that the *degree* of extensification was linked with urban scale, and the findings were further corroborated by ecological analysis of arable weed data from one of the key sites, Tell Brak, north‐east Syria, in the Early Bronze Age (Bogaard, Styring, Ater, et al., 2018). In another study, focusing on early urbanization (or centralization) at early Iron Age hillforts in south‐west Germany (Styring, Rösch, et al., 2017), ecological analysis of arable weed data also revealed an overall trend towards less fertile and less intensively worked soils relative to Neolithic cereal farming. In this case, however, cereal *δ* ^15^N values reflected increased manuring intensity under urbanization, especially of barley, which was used in beer production and elite drinking practices (Styring, Rösch, et al., 2017). The implication is that very different urbanization episodes, such as those in Late Chalcolithic‐Early Bronze Age northern Mesopotamia and early Iron Age south‐west Germany, were characterized by differing degrees of agricultural extensification.

A key variable here, alongside urban scale, may be urban *form*. Although the population density in the closely packed tell cities of northern Mesopotamia is generally assumed to have been between 100 and 150 persons per hectare (Lawrence & Wilkinson, 2015; Wilkinson et al., 2007) and ‘palatial’ Knossos between 200 and 225 persons/ha (Cutler & Whitelaw, 2019), the 100‐ha sprawl of the *Außensiedlung* surrounding the fortified hilltop of the Heuneburg in south‐west Germany contained aggregated farmsteads, each consisting of a fenced compound likely including some arable land (Fernández‐Götz & Krausse, 2013; Kurz, 2010). It seems plausible then that while there would have been little space for cereal cultivation within centres with higher population density, some intensive staple grain production could have been accommodated within the built urban landscape of the less densely occupied Heuneburg. In light of the distinction between these forms of urbanism, we elaborate on the observation by Styring, Charles, et al. (2017) that increasing urban scale was associated with agricultural extensification. We hypothesize that large‐scale *and* high‐density urban forms entailed radical extensification, whereas lower density forms of urbanism, irrespective of scale, allowed more modest extensification, or even ‘true’ intensification, because at least some intensive arable production could be accommodated within the built urban landscape itself.

Here, we aim to test this hypothesis using cereal grain *δ* ^15^N values as a proxy for manuring and agricultural intensity (Bogaard, Styring, Whitlam, et al., 2018; Styring, Rösch, et al., 2017). We incorporate the results of stable isotope studies of cereal assemblages from Neolithic to Bronze/Iron Age settlement sites in northern Mesopotamia, the Aegean and south‐west Germany. The largest urban sites in northern Mesopotamia (Late Chalcolithic 3–4 Tell Brak, EJ II–IV Tell Leilan) and the Aegean (Late Bronze Age Knossos) are examples of densely occupied urban centres with average population densities of 100+ people/ha (Lawrence & Wilkinson, 2015; Whitelaw, 2004). These far exceed that of the urban centre of the Heuneburg in south‐west Germany, whose 100‐ha outer settlement surrounding the much more densely populated fortified hilltop has been estimated to have an average (albeit highly variable) population density of 35 people/ha (Fernández‐Götz & Krausse, 2013; Krausse et al., 2019; Kurz, 2010).

**FIGURE 1:**
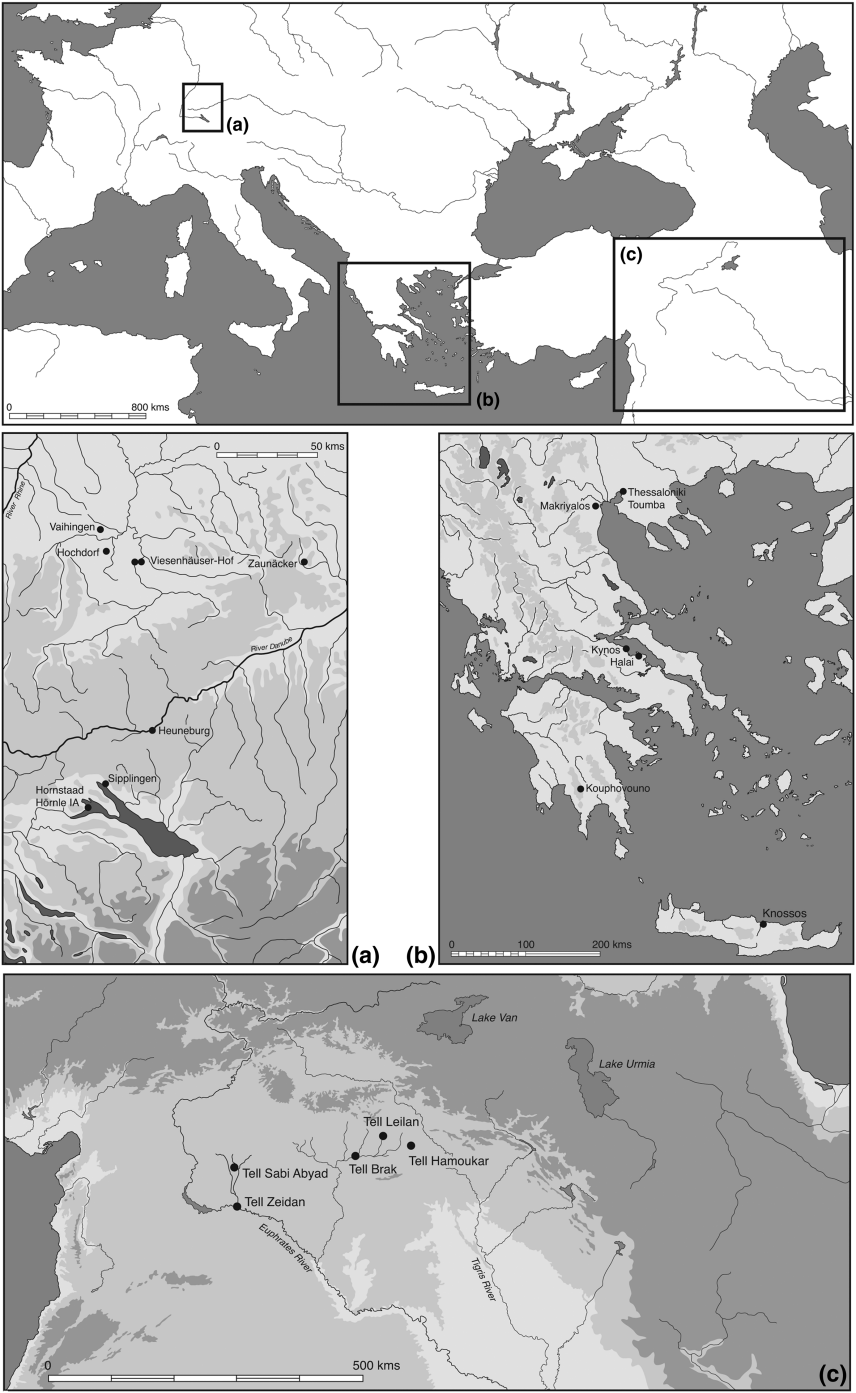
Geographical location of the study areas. Main map: Overview of the study area locations. (a) The location of the archaeological sites in south‐west Germany included in this study. (b) The location of the archaeological sites in Greece included in this study. (c) The location of the archaeological sites in Syria included in this study

Table 1 summarizes the sites, phases and settlement sizes considered here, while Figure 1 shows their geographical distribution. In northern Mesopotamia, the smaller sites comprise the village of Tell Sabi Abyad and the larger (10+ ha) towns of Tell Zeidan and Hamoukar. The larger urban centres of Tell Brak and Tell Leilan fluctuated in size during their long periods of occupation, providing an opportunity to explore how the intensity of farming practice was affected by settlement size in the same location. In the Aegean, smaller settlements include both settlement mound (tell) and extended flat sites smaller than 5 ha and the 28‐ha settlement of Makriyalos enclosed by concentric ditches. Late Bronze Age Knossos represents the largest urban centre in the Aegean, with an estimated extent of 60 ha in the Final Palatial period (mid‐second millennium BC). Cereal grains from earlier, Neolithic, occupation levels at Knossos again provide an opportunity to investigate the effect of the settlement's size on the intensity of farming practice. In south‐west Germany, smaller sites comprise rural settlements smaller than 10 ha dated to the Neolithic and a farmstead and wealthy rural settlement dated to the Early Iron Age. These Iron Age settlements are located close to a hillfort and rich burial mound, respectively, implying special status despite their rural nature. The fortified hilltop settlement of the Heuneburg, with its 100‐ha outer settlement, is the largest urban centre in south‐west Germany included in this analysis.

**TABLE 1:** Details of archaeological sites, including location, chronology, settlement size and sample details

Region	Site	Location (latitude, longitude)	Present‐day annual rainfall (mm)	Archaeological phase	Date (cal BC)	Settlement size (ha)	Site description	Summary of contexts	References for nitrogen isotope values
Northern Mesopotamia	Tell Sabi Abyad	39.09, 36.50	280	Early Pottery Neolithic–Halaf	6,700–5,850	1	Small settlement characterised by a series of mainly rectilinear buildings separated by much open space in the Early Pottery Neolithic. In the Halaf period, a dense agglomeration of multi‐roomed buildings that may have served as collective storage facilities	Domestic occupation deposits including crop storage	Styring, Charles, et al. (2017)
	Tell Zeidan	35.94, 39.08	182	Ubaid–Late Chalcolithic 2	5,300–3,850	12	Large town or regional centre on fertile floodplain, with public buildings, private houses and specialized craft areas	Pyrotechnic features and domestic contexts	
	Tell Brak	36.67, 41.06	363	Late Chalcolithic 2	4,200–3,900	55	Dispersed settlement, including monumental architecture, composed of central mounded area surrounded by a halo of low mounds and flat areas	Mix of workshops, storage, industrial features and monumental buildings	
				Late Chalcolithic 3–4	3,900–3,600	130	A spatially and demographically large urban centre with monumental architecture	Public building, private households and courtyards	
				Late Chalcolithic 4–5	3,600–3,000	45	Settlement on the central mound, comprising mainly domestic buildings and middens	Large house with southern late Uruk ceramics	
				Early Jezireh 0	3,000–2,900	45	An independent city‐state, with domestic buildings and at least one public building	Pit cutting LC4–5 house	
				Early Jezireh III–IV	2,500–2,100	70	An outpost of the southern Mesopotamian Akkadian state, with large administrative buildings, temples, courtyards and industrial features	Domestic quarters within a ‘high status’ household
				Early Jezireh V	2,100–2000	45	A smaller settlement where many of the public buildings from the previous period were converted to private use	Potentially ‘public building’	
	Hamoukar	36.81, 41.96	445	Late Chalcolithic	3,800–3,500	15	Substantial town with evidence for food production on a large scale. Destroyed in 3500 BC, with evidence of violence	Area B: Tripartite buildings, large ovens	
	Tell Leilan	36.96, 41.51	446	Early Jezireh II (Leilan IIId)	2,700–2,600	90	Substantial urban centre with a monumental wall, centralized storerooms, public buildings and a planned street layout	Acropolis northwest public stores; lower town south residential buildings	
				Early Jezireh III (Leilan IIa)	2,600–2,300	90		Lower town south residential buildings	
				Early Jezireh IV (Leilan IIb)	2,300–2,230	90		Lower town south residential buildings; acropolis northwest Akkadian palace	
				Early Jezireh V (Leilan IIc)	2,230–2,200	0.1	Occupation of the urban centre ceased and the only structure was a four‐room courtyard‐centred house that was occupied for around 30–50 years	Acropolis post‐Akkadian four‐room house	
Aegean	Knossos	35.30, 25.16	589	Early Neolithic	7,000–6,450	0.3	Tell settlement that grew from a small hamlet to a large village over the course of the Neolithic	Domestic occupation deposits, possibly including crop storage	This study; Nitsch et al. (2019)
				Late Neolithic	5,500–4,500	1.75–4.5			This study
				Final Neolithic	4,500–3,500	1.75–4.5			
				Late Bronze Age	1,490–1,430	60	Urban settlement centred on palace; the late Minoan II unexplored mansion is part of an elite residential area of the palatial core	Unexplored mansion, storeroom P; elite building; crop storage (pantry) deposits	Nitsch et al. (2019)
	Halai	38.66, 23.19	561	Middle‐Late Neolithic	5,900–4,850	0.7	Small rural settlement	Domestic occupation deposits including crop storage	Vaiglova et al. (2020)
	Kouphovouno	37.06, 22.42	718	Middle Neolithic	5,800–5,500	4	Settlement mound (tell) comprising small houses separated by courtyards and passages	Domestic occupation deposits including crop storage	Vaiglova, Bogaard, et al. (2014); Vaiglova et al. (2020)
				Late Neolithic	5,500–5,000				
	Makriyalos	40.42, 22.59	443	Late Neolithic	5,500–4,950	28	Clusters of semi‐subterranean pit‐dwellings enclosed by a pair of concentric ditches	Domestic occupation deposits	Vignola et al. (2017)
	Thessaloniki Toumba	40.62, 22.97	453	Middle Bronze Age	2,100–1,650	0.5	Compact tell settlement, surrounded by casemate wall in Late Bronze Age	Domestic occupation deposits including crop storage	Nitsch et al. (2017)
				Late Bronze Age	1,375–1,050	0.285
	Kynos	38.72, 23.06	579	Late Bronze Age	1,200–1,050	3	Coastal tell settlement with multiple architectural phases of late Helladic IIIC domestic units	Domestic occupation deposits including crop storage	This study
South‐west Germany	Vaihingen an der Enz	48.93, 8.96	687	Early Neolithic	5,500–5,070	6	Rural settlement of 40–50 longhouses, encircled by an interrupted ditch	Domestic occupation deposits	Fraser et al. (2013)
	Hornstaad‐Hörnle IA	47.69, 9.01	740	Late Neolithic	3,918–3,902	0.21	Lakeshore settlement comprising about 40 houses raised on stilts	Domestic crop stores	Styring, Maier, et al. (2016); Styring, Rösch, et al. (2017)
	Sipplingen Osthafen	47.79, 9.10	893	Late Neolithic	4,000–2,800	1.5	Lakeshore settlement with an estimated maximum population of 750 people	Domestic occupation deposits
	Stuttgart‐Mühlhausen Viesenhäuser Hof	48.84, 9.24	684	Early‐Middle Neolithic	5,500–4,000	8	Partially excavated rural settlement next to a large cemetery	Domestic occupation deposits including crop storage	Styring, Rösch, et al. (2017)
	Eberdingen‐Hochdorf Reps	48.89, 9.00	740	Early Iron Age (La Tène A)	450–400	15	Rural but wealthy settlement of around 40 pit houses and a large ‘mansion’. Rich Halstatt burial mound nearby	Domestic occupation deposits including crop storage and malting pit residue	
	Kirchheim‐Osterholz Ipf‐Zaunäcker	48.87, 10.38	809	Early Iron Age (Halstatt D2–3/La Tène A)	600–400	1.1	Rectilinear farmstead, high quality artefacts and proximity to hillfort of Ipf suggest special status	Domestic occupation deposits	
	Heuneburg	48.09, 9.41	758	Early Iron Age (Halstatt D1‐D3)	600–450	100	Fortified hilltop settlement with evidence for production and trade and an extensive outer settlement of farmsteads enclosed by palisades	Domestic occupation deposits from the Vorburg, the densely settled lower town	

Cereal grain *δ* ^15^N values provide an opportunity to investigate how farming practices related to urbanization processes that manifested themselves in very different forms. Most of the stable isotope data for the cereal assemblages have already been published (see references in Table 1), but we include here new data from the Neolithic sequence at Knossos, Crete (Evans, 1994) and from Late Bronze Age Kynos, central Greece (Dakoronia, 2003, 2010; Kounouklas, 2011).

Moreover, here we take a new, semi‐modular inference approach (Carmona & Nicholls, 2020) to assess the relationship between cereal grain *δ* ^15^N values and settlement size across the three regional data sets. Semi‐modular inference is an improvement on the stepwise multiple imputation approach previously taken by Styring, Charles, et al. (2017) for northern Mesopotamia, because it specifies an optimal rate of information flow between different components of the model (here, the inference of manuring from cereal grain *δ* ^15^N values and the assessment of the relationship between manuring and settlement size). In doing so, semi‐modular inference more fully enables Bayesian inference and addresses the problem of ‘dilution’ in multiple imputation approaches (Knuiman et al., 1998), whereby the apparent effect of interest tends to shrink towards zero.

## 2 METHODS

### 2.1 Determining agricultural intensity using crop isotope values

Crop *δ* ^15^N values largely reflect the nitrogen isotope composition of the soil in which they are grown. This itself reflects the *δ* ^15^N value of nitrogen inputs and the subsequent effects of nitrogen cycling processes (see, e.g., Högberg, 1997 for a review). Application of animal manure, in particular, has been found to increase the *δ* ^15^N values of soil and cereals by as much as 10 ‰ (e.g., Bogaard et al., 2007; Bol et al., 2005; Fraser et al., 2011; Styring, Ater, et al., 2016). The degree to which animal manure increases cereal grain *δ* ^15^N values is variable and relates to the amount and frequency of application, as well as to the type—compost, animal manure and household waste—of organic matter applied (Szpak, 2014). Crop *δ* ^15^N values therefore have the potential to reveal if, and to what extent, soil improvement practices like manuring were employed, whether certain crops were treated differently from others and how such practices changed through time.

Given the number of factors that can influence crop *δ* ^15^N values, Bogaard et al. (2013) categorized manuring intensity into three broad levels—low, medium and high—based on the *δ* ^15^N values of cereal grains grown at long‐term agricultural experiments in Europe. Styring, Charles, et al. (2017) further refined this framework to allow comparison between different geographic regions, by taking into account the fact that aridity can also increase plant *δ* ^15^N values (e.g., Handley et al., 1999). They developed a fitted linear model relating cereal grain *δ* ^15^N values, collated from studies of modern farming regimes across Europe, northern Africa and western Asia, to mean annual rainfall for each manuring level (Styring, Charles, et al., 2017: Figure 2). High manuring intensity was defined as annual manuring of crops at rates equivalent to 30+ tonnes manure ha^−1^; medium manuring intensity as annual or biennial manuring of crops at lower levels (<20 tonnes ha^−1^); and low manuring intensity as no manuring within the last 3+ years. It is these categories that have been most widely adopted in the archaeological literature and which we use in this study.

**FIGURE 2:**
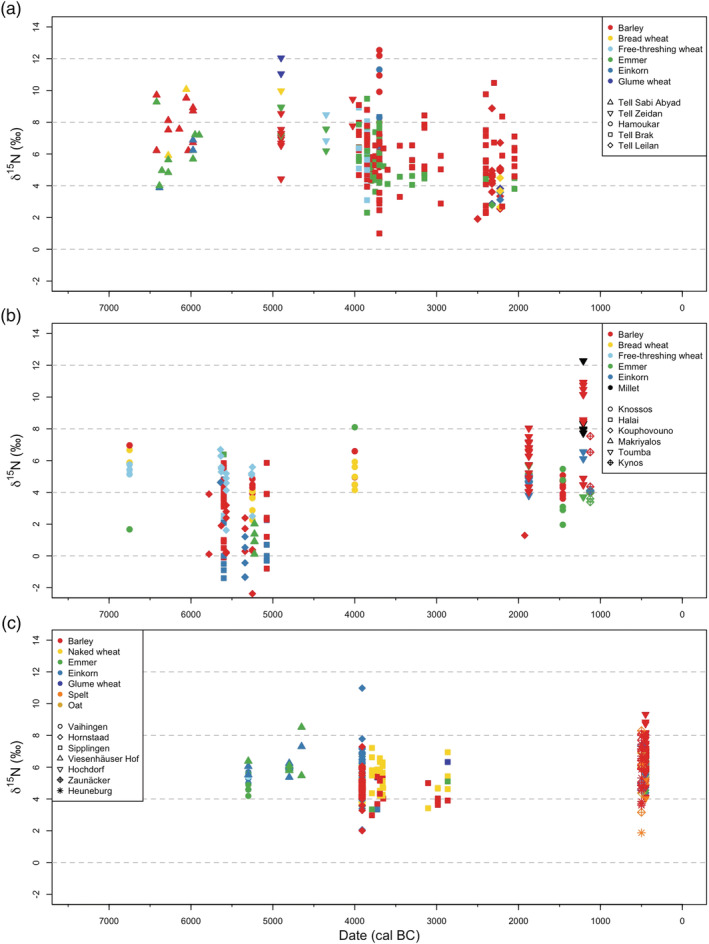
Archaeological cereal grain sample δ^15^N values plotted against date. (a) Cereal grains samples from northern Mesopotamia. (b) Cereal grain samples from the Aegean. (c) Cereal grain samples from south‐west Germany. The shapes of the symbols vary with site, and the symbols are colour coded by crop taxon. Dating of the crop samples is based on stratigraphic relationships to radiocarbon‐dated contexts. More details of the samples are given in Table S1

A number of archaeological studies have now used the *δ* ^15^N values of archaeobotanical remains preserved by charring to reconstruct manuring practices in Europe (Aguilera et al., 2008, 2018; Alagich et al., 2018; Bogaard et al., 2013; Fraser et al., 2013; Gron et al., 2017; Lightfoot & Stevens, 2012; Nitsch et al., 2017, 2019; Styring, Maier, et al., 2016; Styring, Rösch, et al., 2017; Vaiglova et al., 2020; Vaiglova, Bogaard, et al., 2014) and western Asia (Araus et al., 2014; Fiorentino et al., 2012; Styring, Ater, et al., 2016; Styring, Charles, et al., 2017; Vignola et al., 2017). Because intensive manuring characterizes *labour*‐intensive/‐limited agriculture, crop *δ* ^15^N values can act as a proxy for manuring and by extension, the general intensity of agricultural practice. Other forms of intensity can be captured in other ways; for example, soil disturbance through tillage and hand‐weeding can be monitored through the ecology of the associated weed flora, and this is considered elsewhere for the sites in northern Mesopotamia and Germany (Bogaard, Styring, Ater, et al., 2018; Styring, Rösch, et al., 2017) but is not yet feasible for most of the Aegean sites because archaeobotanical weed data are sparse. Water management is potentially another form of intensity and is accessible through crop stable carbon isotope values (e.g., Wallace et al., 2013), but its relevance to our study regions is variable, and strategic planting in wetter soils versus labour input through irrigation works is difficult to assess without preserved evidence of landesque investment.

### 2.2 Statistical background

In their study of changing agricultural intensity with the emergence of urban centres in northern Mesopotamia, Styring, Charles, et al. (2017) used single imputation and Bayesian multiple imputation (Little & Rubin, 2002) to assign manuring levels to the archaeological cereal grain samples and to test for a relationship between these assigned manuring levels and settlement size. Single imputation assigns a single maximum‐likelihood set of manuring levels and then uses this one fixed set of imputed manuring levels to test for an effect due to settlement size. However, we should allow for uncertainty in assigned manuring levels when we estimate settlement‐size effects. Bayesian multiple imputation (BMI) incorporates all important sources of uncertainty—rainfall estimates, assignment of manuring level and the unknown effects of settlement size and date. In contrast to standard Bayesian inference, BMI allows no feedback from settlement‐size effect estimation into manure‐level imputation. This makes it robust to model error but leads to relatively conservative size‐effect estimates.

In this paper, we take a new, semi‐modular inference approach (Carmona & Nicholls, 2020) to assess the relationship between cereal grain *δ* ^15^N values and site‐related parameters across the three regional data sets. Semi‐modular inference occupies a kind of middle ground between standard Bayesian inference and BMI. It measures model error, and this measurement determines an optimal rate of information feedback between settlement‐size effect estimation and manure‐level imputation. This addresses the problem of ‘dilution’ of effects that occurs in multiple imputation approaches (Knuiman et al., 1998). We describe this approach in more detail below (‘determining manuring intensity at archaeological sites’).

### 2.3 Defining site‐ and phase‐related variables: Date, size and rainfall

The date of crop samples is based on their on‐site stratigraphic relationship with radiocarbon‐dated contexts or on regionally established chronologies based on pottery typologies and radiocarbon dates. References for the dating of samples are given in Table S1. Estimates of settlement size are based on field surveys of concentrated surface sherd scatters (as opposed to the continuous and more sparse scatters of small, abraded sherds that have been interpreted as manuring) and targeted excavation for northern Mesopotamia and the Aegean and geophysical prospecting and excavation for south‐west Germany (see Table S1 for references).

In the absence of climatic information derived from high resolution records in close proximity to the archaeological sites, past rainfall estimates are based on present‐day annual rainfall values for each site, which were derived from interpolation of average monthly climate data for 1960–1990 from the WorldClim database (Hijmans et al., 2005). For the sites in northern Mesopotamia, we have used the past rainfall estimates in Styring, Charles, et al. (2017) (Table S1). Styring, Charles, et al. (2017) used the difference between past and present‐day speleothem oxygen isotope (*δ* ^18^O) values at Soreq Cave, Israel, and the present‐day calibration relationship between speleothem *δ* ^18^O values and rainfall (a 1‰ decrease in the *δ* ^18^O value of precipitation is equivalent to an increase in annual rainfall of about 200 mm; Bar‐Matthews & Ayalon, 2004) to adjust present‐day annual rainfall at each of the archaeological sites and thus estimate past rainfall at 200‐year intervals. For the sites in Greece and southwest Germany, we referred to Figs. 6 and 7 in Mauri et al. (2015), which model summer and winter rainfall anomalies relative to AD 1850 at thousand‐year intervals based on pollen data. For both summer and winter, we looked for the most extreme monthly rainfall deviations in the general region of each archaeological site for the relevant time slice and multiplied these by the number of months (six in summer and six in winter). We then summed the summer and winter anomaly values to give a minimum and maximum deviation from modern‐day rainfall. The past rainfall value is an average of the two. The uncertainty associated with these estimates is accounted for in the statistical models.

### 2.4 Determining the *δ* ^15^N values of archaeological cereal grain samples

The majority of cereal grain sample *δ* ^15^N values used in this study have been published previously, and thus we refer to these publications in Table 1. The *δ* ^15^N values of cereal grain samples from Kynos and some from Neolithic Knossos have not yet been published, and further details of their preparation are given here. These methods are largely consistent with those used to prepare and determine the *δ* ^15^N values of the other cereal grain samples from northern Mesopotamia, the Aegean and south‐west Germany.

Cereal grains from Kynos and Neolithic Knossos were subsampled from grain‐rich deposits including storage contexts. For a given species within each context, between four and 12 grains were homogenized per sample. Fourier‐transform infrared spectroscopy analysis of a subset of samples ruled out any significant sources of contamination (cf. Vaiglova, Snoeck, et al., 2014), and so visible surface contaminants, such as adhering sediment, were removed by gentle scraping and grains were crushed using an agate mortar and pestle. Samples were weighed out to 2–4 mg for nitrogen isotope value determinations, which were carried out separately from carbon isotope value determinations due to the low nitrogen content of the samples.

Isotope value determinations were made on a Sercon 20–22 isotope ratio mass spectrometer coupled to a Sercon GSL elemental analyser operating in continuous flow mode at the Research Laboratory for Archaeology and the History of Art, University of Oxford, UK. Raw and drift‐corrected isotope ratios were calculated against an internal alanine standard; *δ* ^15^N values normalized to the AIR scale were calculated against two bracketing reference materials: Caffeine‐2* (U. Indiana, *δ* ^15^N −2.9 ± 0.03 ‰) and IAEA‐N2 (*δ* ^15^N 20.3 ± 0.2 ‰). Normalization and measurement uncertainty were calculated using the approximation method reported by Kragten (1994). This and all other statistical calculations were performed using the programming language R (3.2.4). The average measurement uncertainty for *δ* ^15^N values was 0.27 ‰, with a range from 0.17 to 0.34 ‰. The *δ* ^15^N values of the archaeological crop samples from Kynos and Knossos are given in Table S1, normalized to the standard values. The crop isotope results are also reported corrected for the minor effect of charring on *δ* ^15^N values (Nitsch et al., 2015). Full details of the statistical analysis (including R files) are available in Supporting Information and Supplementary Code.

## 3 DETERMINING MANURING INTENSITY AT ARCHAEOLOGICAL SITES

Table S1 presents the archaeological cereal grain *δ* ^15^N values from sites in northern Mesopotamia, the Aegean and south‐west Germany, including previously published data and new determinations on samples from Knossos and Kynos, Greece. Figure 2 plots these archaeological cereal grain *δ* ^15^N values against date, according to geographic region.

Following Styring, Charles, et al. (2017), we assign manuring levels to the archaeological cereal grain samples, derived from the fitted linear model regressing modern cereal grain *δ* ^15^N values against rainfall data (Styring, Charles, et al., 2017, Figure 2) and based on the archaeological cereal grain sample *δ* ^15^N value and estimated rainfall for each archaeological site and phase. This approach takes the effect of aridity on cereal grain *δ* ^15^N values into account, providing a more accurate and conservative estimate of manuring intensities for the archaeological cereal grain samples than direct comparison with the *δ* ^15^N values of modern cereals grown in agricultural experiments in temperate Europe (Bogaard et al., 2013; Fraser et al., 2011).

We assess the relationship between manuring and settlement size using three statistical approaches: single imputation, Bayesian multiple imputation and semi‐modular inference (see Supporting Information). We present the results of each approach in turn, from simplest to most complex.

In single imputation, each archaeological cereal sample is first assigned its most probable manuring level using the fitted linear model relating modern cereal grain *δ* ^15^N values to mean annual rainfall for low, medium and high manuring levels. Secondly, the assigned manuring levels are regressed against settlement size to test for an association between the two using mixed‐effects proportional odds models. A large number of proportional odds models were considered, incorporating varying combinations of the co‐variates site, size and date and their transformations (see Section S3 for more detail). For both the data sets from northern Mesopotamian and the Aegean, the most successful models incorporated the variable size, whereas for the data set from south‐west Germany, the best performing models incorporated random effects of site, without size. Date was found to have no effect in all three data sets. A Wald test indicates a negative effect of settlement size on manuring level in the Aegean (estimated coefficient = −0.508) that is significant at level 0.05 (*p* = 0.0297). This effect is similar to the results previously reported for northern Mesopotamia (estimated coefficient = −0.464; *p* = 0.0069) (Styring, Charles, et al., 2017). In contrast to these two regions, there is no evidence that settlement size has an effect on manuring level in south‐west Germany. If settlement size is included in the model, it is found to have a positive effect on manuring level (estimated coefficient = 0.250), but this is *not* significant (*p* = 0.50). The single imputation approach does not take into account uncertainty relating to the assignment of manuring level and to past rainfall ranges at the archaeological sites and so may overstate the strength of relationships between manuring level and settlement size.

Next, we use Bayesian multiple imputation to assign manuring levels to the archaeological cereal grain samples and to simultaneously test for a relationship between these assigned manuring levels and site‐related parameters such as size and date, following Styring, Charles, et al. (2017) (see Section S4 for more detail). In the first ‘imputation’ stage of multiple imputation, the probability of each cereal grain sample having each manuring level value is imputed. This is derived from the regression between modern cereal grain *δ* ^15^N values and rainfall data and is based on the archaeological cereal grain sample *δ* ^15^N value and estimated rainfall for each archaeological site and phase (accounting for uncertainty). In the second ‘analysis’ stage, multiple data sets of assigned manuring levels (each slightly different due to the uncertainty associated with assigning a manuring level) are sampled from the manuring level probability distribution that was derived in the first stage. We regress the assigned manuring level data against settlement size and test for a negative (one‐sided) effect. We expanded the proportional odds model used in the single imputation approach and used by Styring, Charles, et al. (2017) in their Bayesian multiple imputation approach by incorporating date, as well as settlement size, as fixed variables and site as a random variable. This makes the Bayesian analysis self‐contained and allows us to explicitly test for an effect of date. The final estimated posterior odds (equivalently here, the Bayes factor) are 5.3 to 1 in favour of a negative effect of settlement size on the manuring level of cereal grains from northern Mesopotamia and 2.3 to 1 in favour of a negative effect of settlement size on the manuring level of cereal grains from the Aegean. In contrast, for south‐west Germany, the Bayes factor is 0.8, reflecting very weak support for a positive effect of settlement size on the manuring level of cereal grains.

**FIGURE 3:**
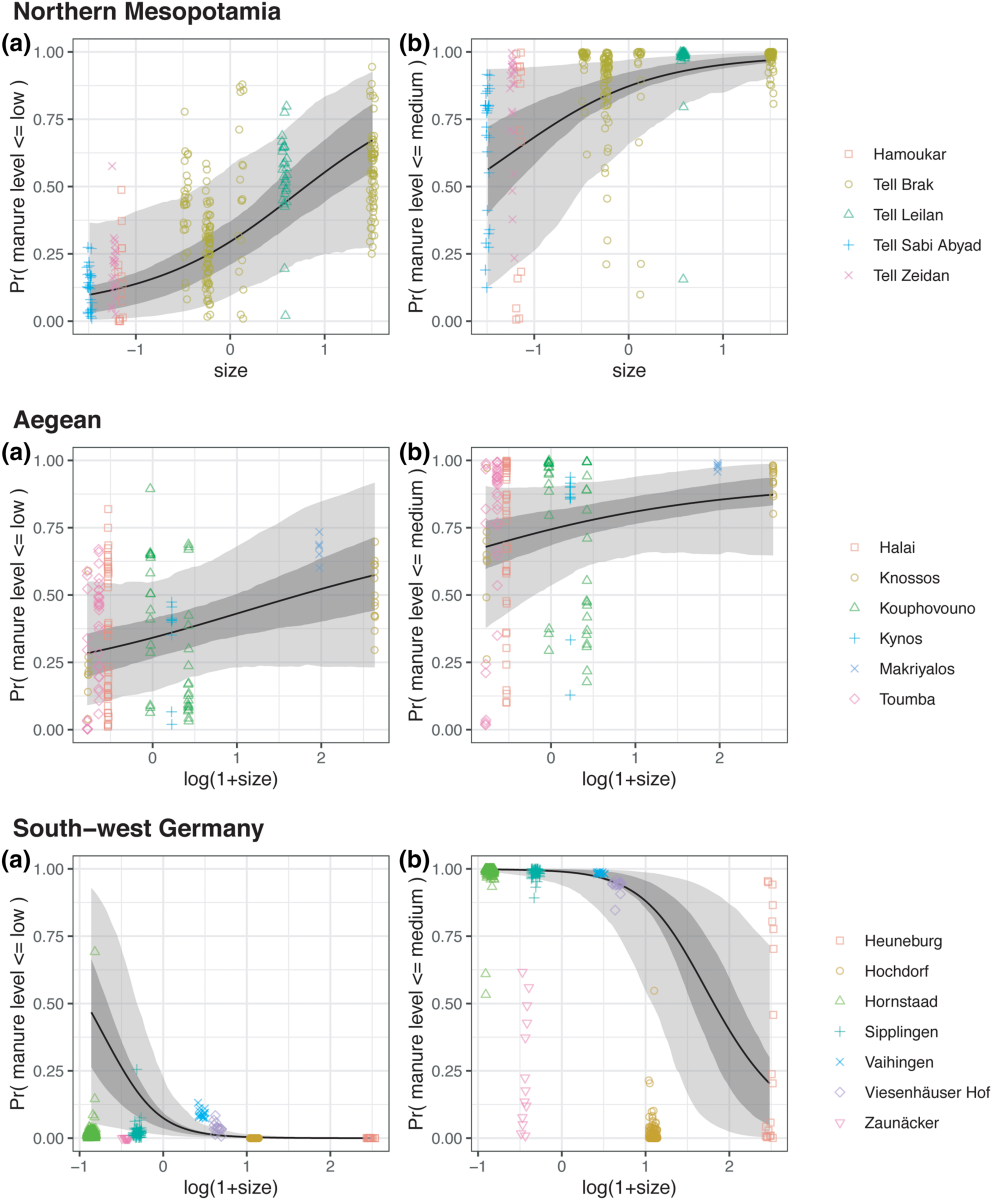
The posterior probability (*y*‐axis) that a given archaeological cereal grain sample has a manuring level equal to *m* or lower is plotted against site size (*x*‐axis). (a) Manuring level *m* = low. (b) Manuring level *m* = medium. Shaded areas give 50% and 90% credible intervals for the probability. Each row corresponds to a data set: northern Mesopotamia (top), Aegean (middle) and south‐west Germany (bottom). Colours and symbols distinguish different sites

As noted in the Introduction, multiple imputation suffers from a problem of ‘dilution’, meaning that estimated Bayes factors are conservative measures of the strength of evidence. As a third approach, therefore, we use semi‐modular inference to specify an optimal rate of information flow between components of the model (here, the inference of manuring level from cereal *δ* ^15^N values, and the assessment of the relationship between manuring level and settlement size), enabling a fully Bayesian approach. In this way, we maximize the predictive performance of the model (see Section S5 for more detail). Using this approach, the Bayes factor is 65.4 to 1 in favour of a negative effect of settlement size on the manuring level of cereal grains from northern Mesopotamia and 6.2 to 1 in favour of a negative effect of settlement size on the manuring level of cereal grains from the Aegean. The Bayes factor of 0.2 for south‐west Germany is evidence for a positive effect of settlement size on the manuring level of cereal grains. These refinements inform Figure 3, which shows the probability of an archaeological cereal grain sample having a manuring level of *m* (low or medium) or lower, plotted against settlement size, for each of the three regional data sets. It is apparent that, although these relationships are positive for northern Mesopotamia and the Aegean, indicating a decrease in manuring intensity as settlement size increases, the picture for south‐west Germany is very different, showing no relationship or even an increase in manuring intensity as settlement size increases.

## 4 ASSESSING REGIONAL PATTERNS IN THE RELATIONSHIP BETWEEN CEREAL MANURING INTENSITY AND URBAN FORM

The single and multiple imputation and semi‐modular inference approaches all show that the probability that cereals received low‐moderate levels of manure tends to rise with increasing settlement size in northern Mesopotamia and the Aegean (Figure 3). This is consistent with extensification of cultivation as settlement size increased, likely concomitant with an increase in population (e.g. Neolithic‐Bronze Age Knossos, Isaakidou, 2008). The negative relationship between manuring intensity and settlement size was already shown by Styring, Charles, et al. (2017) for Neolithic and Early Bronze Age sites in northern Mesopotamia, and this study demonstrates the same relationship for Neolithic and Bronze Age sites in the Aegean. Use of semi‐modular inference to test for a significant effect of settlement size on manuring intensity has been found to be a more robust way of examining this relationship, mitigating the ‘diluting’ influence of uncertainty on effect size encountered using the Bayesian multiple imputation.

The largest settlements in northern Mesopotamia and the Aegean (LC 3–4 Tell Brak, EJ II–IV Tell Leilan, Late Bronze Age Knossos) are examples of densely occupied urban centres. Although there was likely variation in occupation density across the overall footprint of these sites—for example, Tell Brak, where sherd scatters are concentrated on a central mound, surrounded by a lower density halo of sherds and outlined by a further ring of mounds at its edge (Ur et al., 2011)—their average population density likely exceeded 100+ people/ha. In these densely packed settlements, there would have been little space for cereal cultivation *within* the settlement itself—particularly not on a scale sufficient to supply the surplus produce found in large‐scale storage complexes at these sites and to feed their inhabitants—and arable fields would therefore have expanded outwards beyond the main settlement area. Indeed, scatters of abraded sherds proposed to originate from household waste that was spread onto fields and ‘hollow ways’—radiating tracks resulting from confining animal movement between cultivated fields—have been used to identify the past extent of arable land (Wilkinson et al., 2010) and archaeological surveys have demonstrated very little settlement within 3–4 km of major sites in northern Mesopotamia (Wilkinson, 2003, p. 122). There is thus a sharp distinction between urban and rural environments in these regions, with fields progressively further from the urban core having received decreasing levels of organic matter/household waste due to the effort required to transport this heavy and bulky resource from its source (Halstead, 2014, pp. 216–219). As Styring, Charles, et al. (2017) note, although effort expended in manuring plots may have increased *overall* at larger settlements, the bulk of the increase in cereal production must have come from expansion of these less intensively manured plots, located at a greater distance (>500 m cf. Halstead, 2014, pp. 216–219) from the main habitation areas.

In contrast to northern Mesopotamia and the Aegean, the single and multiple imputation and semi‐modular inference approaches all show that in south‐west Germany, there is *no* evidence for a decrease in manuring intensity as settlements grew in the Early Iron Age. Rather, the manuring rate seems to have been maintained or even increased in an attempt to moderate the decrease in soil fertility evidenced by weed ecological data, presumably due to long‐term exploitation (Styring, Rösch, et al., 2017). The farmsteads that characterized the outer settlement of the Heuneburg had some space for cultivation of cereals, and the close proximity of cereal fields to households would have enabled continued high manuring rates. The presence of fences around these farmsteads (Krausse et al., 2016) also attests to the penning of livestock, whose manure would have ensured a supply of organic matter to the fields. There is also growing evidence for dispersed settlement beyond the 100 ha currently attributed to the outer settlement of the Heuneburg itself, attesting to occupation of and food production in the wider hinterland (Hansen et al., 2017; Krausse et al., 2019). The findings of this study thus support our hypothesis that low‐density forms of urbanism allowed at least some intensive staple grain production to be accommodated within the built urban landscape itself and its nearby surroundings. As such, although weed ecological evidence suggests there was modest expansion of cultivated areas, manuring intensity could be maintained (Styring, Rösch, et al., 2017).

This finding adds another dimension to the thesis advanced by Fletcher (1995) that high population density constrained possibilities for interaction and communication, thereby imposing a limit on settlement growth. Inhabitants of densely occupied sites would have had restricted access to nearby land for cultivation of cereals, preventing the continuation of intensive cultivation practices characteristic of smaller settlements. Producers would have been less able to interact with and were physically separated from agricultural land, precipitating the transition to an extensification strategy. By contrast, in low‐density urban centres, a mosaic of settlement and intensively managed cultivation areas could expand indefinitely, with the potential to form vast agro‐urban landscapes (Fletcher, 1995, p. 92). To date, water availability has been cited as the defining influence on urban form (e.g., Scarborough & Lucero, 2010) because the main focus has been on (semi‐)tropical examples of dispersed urban centres, including the notable examples of the ancient Khmer capital, Angkor, in Cambodia (Fletcher et al., 2015) and the Mayan city of Tikal in Guatemala (Isendahl, 2012). Our study demonstrates that dispersed settlement also enabled the interaction between producers and arable land to be maintained and intensive agricultural practices such as manuring to persist (cf. Isendahl & Smith, 2013; Moore, 2017). It should be reiterated here that we make the distinction between cereal cultivation and that of other crops, such as pulses. In fact, there is isotopic evidence from Late Bronze Age Knossos that pulses were grown in different conditions from cereals, possibly on small ‘allotment’ plots that could have been accommodated within or on the margins of the urban sprawl (Nitsch et al., 2019).

Extensive agriculture is an inflexible system that presupposes marked economic inequality: In a pre‐mechanized/pre‐industrial farming system, cultivation of cereals on large tracts of land places a significant demand for highly seasonal animal (for ploughing) and human (for harvesting) labour, beyond the reach of individual households, makes intensive manuring impracticable (both because production of manure on an adequate scale requires large numbers of animals and because of the difficulty of transport), erodes crop diversity and requires institutions that enable very strict control of ownership of land, or at least its produce (e.g., Halstead, 1995). As a result, once extensive agriculture became established, communities were effectively locked into this mode of production, and institutionalized social inequality could persist for millennia (Bogaard et al., 2019). Although the urban centres of northern Mesopotamia and the Aegean were relatively long lived, there was a millennial‐scale cycle of booms followed by busts in Mesopotamia (Lawrence & Wilkinson, 2015; Ur, 2010), which arguably were to some extent tied to the [in]ability of aggregated centres to sustain an adequate food supply without compromising long‐term soil fertility and thus productivity, particularly in the face of climate change (Lawrence et al., 2016, 2021). A further dimension of this fragility was dependence of high‐density urban centres on surplus extracted from a wider hinterland of smaller settlement; this is well evidenced in both northern Mesopotamia and at Final Palatial Knossos (Whitelaw, 2019; Wilkinson, 1994).

In more intensive farming systems, like south‐west Germany, *the farming system itself* was robust to political change. Intensive manuring practice continued from the Neolithic into the Early Iron Age (Styring, Rösch, et al., 2017), whereas the phenomenon of these first ‘urban’ centres (or *Fürstensitze*) was unstable; the Heuneburg, for example, only lasted for two or three generations (Fernández‐Götz & Ralston, 2017; Krausse, 2008; Krausse et al., 2016). If political power depended on control over land and the means of production (Gosden, 1985), then perhaps the fact that agriculture was less land‐limited means that hold over power was less stable and more vulnerable to other, external, factors. As such, the first ‘urban’ centres of Central Europe were relatively short lived, but aspects of the farming systems dating back thousands of years persisted. Modest agricultural extensification between the Neolithic and Iron Age has also been evidenced in the Rhineland, Germany, where there is no corresponding evidence for centralization in the form of *Fürstensitze* (Hamerow et al., n.d.). This demonstrates that the agrarian system that developed through to the Iron Age was ultimately more sustainable than the power relations it enabled.

Moreover, much in the same way that agricultural intensification as a predetermined response to population growth (Boserup, 1965) has been largely disproved in Europe and western Eurasia, based on evidence for intensive cultivation and manuring from the very beginnings of farming (Bogaard, 2004; Bogaard et al., 2013; Styring, Ater, et al., 2016), this study demonstrates that *radical* agricultural extensification is not a prerequisite of centralization. Just as there was no inexorable ‘march of progress’ towards the type of nucleated centres seen in western Asia but rather a burgeoning of different urban forms (Fernández‐Götz et al., 2014; Fletcher, 2019; Yoffee, 2005), there was no unilinear trajectory towards agricultural extensification.

## 5 CONCLUSIONS

At a time when urban centres are home to the majority of the world's population, it is crucial to understand the interplay between food production and urbanization from a long‐term perspective. From an archaeological standpoint, the relatively labour‐intensive agriculture that characterized the small rural and low‐density urban sites in this study was more socially flexible and adaptable than expansive agriculture reliant on huge swathes of cultivated land, much of it at a distance from the nucleated centres of northern Mesopotamia and the Aegean. Radically extensive agriculture locked high‐density urban societies into long‐term cycles of high wealth inequality that were vulnerable to dramatic collapse, such as occurred in the eastern Mediterranean at the end of the Bronze Age (Cline, 2021). We suggest that these agrarian regimes resemble persistent but maladaptive ecological systems that fall into a ‘rigidity trap’, as outlined by Holling et al. (2002). Our study shows how a practical understanding of cereal production in past agrarian systems sheds new light on power relations associated with different urban forms. Our results underline the importance of tracing land acquisition practices and their consequences for contemporary societies, including transnational ‘land grabs’ in the Global South, for example (Li, 2015).

Crop nitrogen isotope values and use of a regression model that takes into account the effect of rainfall offer a ‘common currency’ with which to compare the intensity of agricultural practices in discrete geographic regions and [pre]historic periods. We hope that the application of this approach in other regions of the world (e.g., Styring et al., 2019) will help to provide a more nuanced insight into agricultural *practice* and how it intersected with politics and society more broadly over the *longue durée*. Alongside focus on the threat to agricultural sustainability posed by loss of *species* diversity, there needs to be a parallel discussion of how past agrosystems maintained a range of ecological niches if we are to maintain and promote biodiversity in the future (e.g., FAO, 2016).

## Supporting information


**Table S1.** Carbonized cereal grain δ^15^N values from Neolithic, Bronze Age and Iron Age sites in northern Mesopotamia, the Aegean and south‐west Germany

**Figure S1:** Key relations in the modern Data. Left: Positive relation between Nitrogen Isotope (normd15n, *y*‐axis) and manure_level (*x*‐axis). Right: Negative relation between normd15n (*y*‐axis) and log(rainfall) (*x*‐axis), with point shapes and colours distinguishing different manure levels.
**Figure S2:** Negative relation between levels of Nitrogen isotope and (log) rainfall. Colour indicates a dataset. Solid lines regress these two variables, separately for each dataset.
**Figure S3:** Relation between levels of Nitrogen isotope and site size for the three archaeological datasets. Solid lines regress these two variables.
**Figure S4:** Observed normd15n‐values coloured by imputed manure level for the three archaeological datasets. Each panel shows the relation between normd15n and log(rainfall); each point corresponds to a single cereal grain‐sample. Modern(Archaeological) data plotted with open(filled) circles. Colours correspond to manuring level (red = low, green = medium, blue = high). For the modern data the manuring levels are known, for the Archaeological data, these are the manure_level.imputed‐values.
**Figure S5:** Posterior distribution of γ, the effect due to size on manuring levels in the archaeological data.
**Figure S6:** Estimated predictive performance measured by ELPD (*y*‐axis) as a function of the control parameter η (*x*‐axis) under SMI for each of the three datasets (see panel titles). Vertical lines give the optimal values η = η* of the control parameter for each dataset.
**Figure S7:** Posterior mean and credible intervals of γ under Semi‐Modular Inference. The y‐axis correspond to the value of, while the x‐axis corresponds to values of the degree of inuence η ∈ [0; 1].
**Figure S8:** Posterior distribution of γ under SMI for the optimal degree of inuence, η*.
**Figure S9:** The posterior probability (*y*‐axis) that a given archaeological cereal grain sample has a manuring level equal *m* or lower is plotted against site size (*x*‐axis). (a) Manuring level *m* = low. (b), Manuring level *m* = medium. Shaded areas give 50% and 90% credible intervals for the probability. Each row corresponds to a dataset: nmeso (top), aegean (middle), and swgermany (bottom). Colours distinguish different sites.
**Figure S10:** Pairwise relation between key variables in the modern data
**Figure S11:** Pairwise relation between key variables in the archaeological data from Northern Mesopotamia (nmeso)
**Figure S12:** Pairwise relation between key variables in the archaeological data from the Greece (aegean)
**Figure S13:** Pairwise relation between key variables in the archaeological data from the Germany (swgermany)
**Figure S14:** Trace plot of the Monte Carlo samples for the main parameters in the model.
**Figure S15:** Heatmap with correlations of the principal parameters in the model (gamma_po_1 corresponds to γ, the effect due to size in PO, whereas gamma_po_2 is the effect due to date, denoted by τ in the text.)

## Data Availability

The data that supports the findings of this study are available in the Supporting Information of this article.
