# Supplementary material for: Urban form and scale shaped the agroecology of early ‘cities’ in northern Mesopotamia, the Aegean and Central Europe
Source: J Agrar Chang. 2022 May 31;22(4):831–54. doi: 10.1111/joac.12497 (PMC9580239; doi:10.1111/joac.12497)
Supplement: Supplementary file 2 — Figure S1: Key relations in the modern Data. Left: Positive relation between Nitrogen Isotope (normd15n, y‐axis) and manure_level (x‐axis). Right: Negative relation between normd15n (y‐axis) and log(rainfall) (x‐axis), with point shapes and colours distinguishing different manure levels. Figure S2: Negative relation between levels of Nitrogen isotope and (log) rainfall. Colour indicates a dataset. Solid lines regress these two variables, separately for each dataset. Figure S3: Relation between levels of Nitrogen isotope and site size for the three archaeological datasets. Solid lines regress these two variables. Figure S4: Observed normd15n‐values coloured by imputed manure level for the three archaeological datasets. Each panel shows the relation between normd15n and log(rainfall); each point corresponds to a single cereal grain‐sample. Modern(Archaeological) data plotted with open(filled) circles. Colours correspond to manuring level (red = low, green = medium, blue = high). For the modern data the manuring levels are known, for the Archaeological data, these are the manure_level.imputed‐values. Figure S5: Posterior distribution of γ, the effect due to size on manuring levels in the archaeological data. Figure S6: Estimated predictive performance measured by ELPD (y‐axis) as a function of the control parameter η (x‐axis) under SMI for each of the three datasets (see panel titles). Vertical lines give the optimal values η = η* of the control parameter for each dataset. Figure S7: Posterior mean and credible intervals of γ under Semi‐Modular Inference. The y‐axis correspond to the value of, while the x‐axis corresponds to values of the degree of inuence η ∈ [0; 1]. Figure S8: Posterior distribution of γ under SMI for the optimal degree of inuence, η*. Figure S9: The posterior probability (y‐axis) that a given archaeological cereal grain sample has a manuring level equal m or lower is plotted against site size (x‐axis). (a) Manuring level m = low. (b), Manuring level [file JOAC-22-831-s001.pdf]

Statistical supplement

Chris U. Carmona & Geoff K. Nicholls

Department of Statistics
University of Oxford
Oxford, UK

1 Introduction

This document is the statistical supplement to "Urban form and scale shaped the agroecology of early cities in northern Mesopotamia, the Aegean and central Europe". We test for an effect due to site size on manure level in the archaeological sites presented in the main paper. We consider new datasets from two archaeological contexts for this test: one from Early Neolithic-Late Bronze Age Greece (the `aegean` data), and a second from Early Neolithic-Early Iron Age south-west Germany (the `swgermany` data). The results given in section 5.3 are reported in the main paper. Our analysis follows the supplement to Styring et al. (2017) in substance. Those authors test for the same effect in similarly structured archaeological data from urban centres in northern Mesopotamia. We use methods from that source throughout this document. However, we improve on the statistical methodology of Styring et al. (2017) in the section 5.

The document is organised as follows. In section 2 we summarise the two new archaeological datasets and the modern dataset we use for calibration. In section 3 we present a *single imputation* analysis, which introduces the statistical models in a simple inferential framework. In section 4 we repeat the test using *Bayesian Multiple Imputation* (BMI), which accounts for uncertainty in the imputation. Up to this point we are using the same methods as Styring et al. (2017) [1]. One weakness of multiple imputation, noted by Styring et al. (2017), is that it usually gives conservative estimates for the strength of any effect. The effect is "diluted" in the sense of Knuiman et al. (1998) due to "imputation noise".

This could be ignored by Styring et al. (2017) as the effect-size of interest remained significant despite dilution. In section 5 we repeat the analysis using *Semi-Modular Inference* (SMI) (Carmona and Nicholls, 2020). This method is closely related to BMI. It measures model misspecification and down-weights but does not remove misspecified model components at the imputation stage. Down-weighting reduces dilution and gives a more accurate measure of evidence than BMI.

Our analyses show a clear (negative) effect due to site size on manure-level in the `nmeso` and `aegean` data, and a potential positive effect in the `swgermany` data. Along the way we reproduce the results of Styring et al. (2017) using a completely independent code base and get good agreement. The results in this supplement can be reproduced using the accompanying scripts in `R`. Data and functions are bundled in the `agricurbayes` package.

```
> require("agricurbayes")
> arc_datasets = c("nmeso","aegean","swgermany")
> agricurb_data <- agricurbayes::get_agricurb_data( arc_datasets=arc_datasets,
+                                                    vars_scale_int_0_1=NULL )
> manure_levels = c("low","medium","high")
> agricurb_data = agricurb_data %>%
+   dplyr::mutate( log_size=log(1+size) ) %>%
+   dplyr::mutate( log_rainfall=log(1+rainfall) ) %>%
+   dplyr::mutate( manure_level=factor(manure_level, levels=manure_levels, ordered=TRUE) ) %>%
+   dplyr::mutate( category=factor(category, levels=c("barley","wheat")) ) %>%
+   dplyr::mutate( dataset=factor(dataset, levels=c("nmeso","aegean","swgermany","modern")) )
```

[1] Our model for Bayesian Multiple Imputation differs slightly from Styring et al. (2017), as we incorporate the effect due to date in the `PO` module (See section 4). The conclusions and evidence for extensification remain unchanged.

2 Data description

2.1 Modern calibration data

The modern data consist of agricultural records gathered in experimental settings in which manure level is an independent variable and nitrogen isotope ratio is the measured outcome. This modern calibration dataset is the same as the one used by Styring et al. (2017). The dataset has observations on 268 grain samples. Six variables are of interest here.

normd15n Measured cereal grain-sample nitrogen isotope ratio.

manure_level The level of manuring treatment the cereal grain-sample received. A 3 level ordinal variable with levels “low”, “medium” and “high”.

site : The name of the site where the grain-sample was gathered. A 19-level categorical variable.

Year : The year in which the grain-sample was gathered.

category : The grain-sample cereal species. A 2-level variable with levels “barley” and “wheat”.

rainfall : annual rainfall estimate for the cereal grain sample site, obtained by interpolation of average monthly local climate data for 1960 - 1990, taken from the WorldClim database.

The data were gathered in experiments conducted under different conditions at different times at different sites. The manuring treatments vary qualitatively and quantitatively from site to site. These manure-level records were mapped to a 3-level ordinal scale with levels low, medium and high. In fig. 10 of appendix A we plot pairwise relations between key variables in the modern data. In particular, note the positive association between Nitrogen Isotope (`normd15n`) and Manure levels (`manure_level`), and the negative relation between `normd15n` and rainfall levels. These are further illustrated in fig. 1.

```
> mod.dat <- agricurb_data %>%
+   dplyr::filter( dataset=="modern" )
```

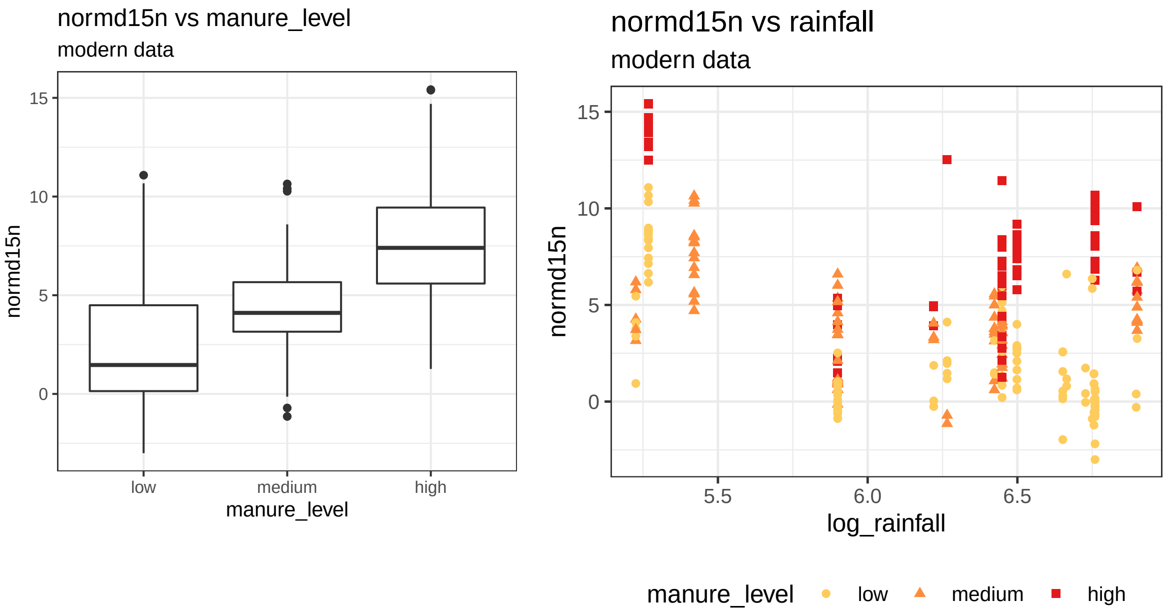

Figure 1: Key relations in the modern Data. Left: Positive relation between Nitrogen Isotope (`normd15n`, y-axis) and `manure_level` (x-axis). Right: Negative relation between `normd15n` (y-axis) and `log(rainfall)` (x-axis), with point shapes and colours distinguishing different manure levels.

2.2 The Archaeological data

The `aegean` and `swgermany` datasets contain observations on 181 and 435 grain-samples respectively. The variables are the same as those used in Styring et al. (2017) in the Mesopotamian context. We include the Northern Mesopotamia dataset (`nmeso`, 269 observations) studied in Styring et al. (2017) for comparison in all analyses.

normd15n : measured grain-sample nitrogen isotope ratio.

site : site name for sample-context. A categorical variable with a level for each site ID, (5 levels for `nmeso`, 6 for `aegean`, and 9 for `swgermany`).

phase : phase name for grain-sample context. A categorical variable with a level for each phase ID, giving the temporal context of the sample (14 levels for `nmeso`, 31 for `aegean`, and 5 for `swgermany`).

date : date of grain-sample context.

size : size of context-site in hectares at sample-context date.

category : grain-sample species. A 2-level categorical variable with levels "barley" and "wheat".

min_rainfall, max_rainfall : upper and lower bounds on rainfall at grain-sample context at context date.

rainfall : estimated annual rainfall at sample-context at context-date.

Note the negative relation between `normd15n` and `log(rainfall)` in all datasets in fig. 2. In contrast the relation between `normd15n` and site `size` shown in fig. 3 varies across datasets. For the `nmeso` and `aegean` datasets this relation is negative, whereas for the `swgermany` dataset it may be slightly positive, if there is any relation at all. In figs. 12 and 13 in appendix A we plot pairwise relations between all key variables in the `aegean` and `swgermany` datasets.

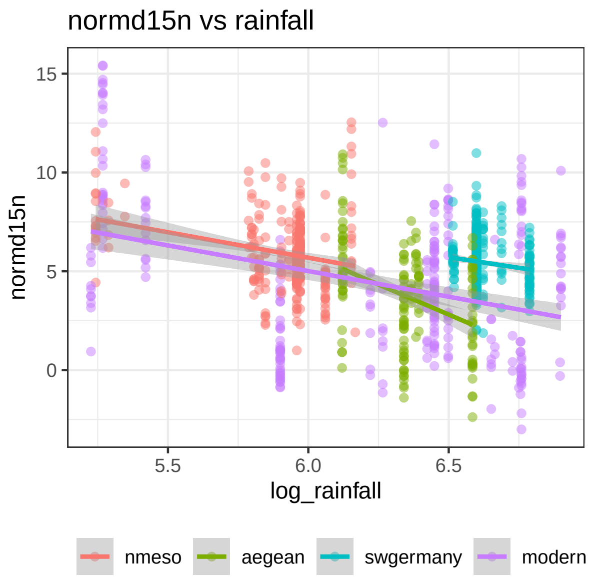

Figure 2: Negative relation between levels of Nitrogen isotope and (log) rainfall. Color indicates a dataset. Solid lines regress these two variables, separately for each dataset.

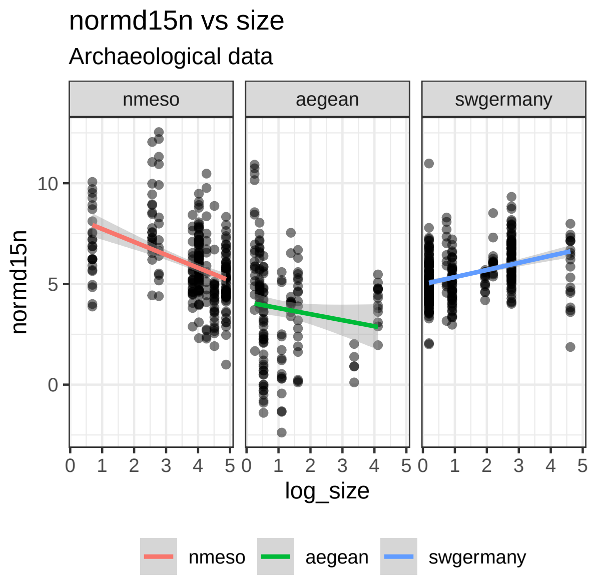

Figure 3: Relation between levels of Nitrogen isotope and site size for the three archaeological datasets. Solid lines regress these two variables.

3 Single Imputation Analysis

In this section we use single imputation to test for an effect due to site size on manuring levels. The aim is to introduce the reader to the models and reasoning in a simple inferential setting. The results here give a sanity check for the more sophisticated analysis in the sections to follow.

In this simple analysis we use the `rainfall` values in the Archaeological data. These should be seen as unreliable compared to the more conservative upper and lower bounds `max_rainfall` and `min_rainfall`. We return to this in sections 4 and 5. Our analysis in this section follows Section 3 of the statistical supplement to Styring et al. (2017).

Our plan is (Step 1) use the `modern` dataset to capture the relation between `manure_level` and `normd15n`. Then (Step 2), invert that relation to estimate the missing `manure_level`-values in the archaeological data from their observed `normd15n` values. This determines `manure_level.imputed` values for the archaeological data. Finally (Step 3), we model the relation between the `manure_level.imputed`-values and `size`, the size of the site associated with the grain-sample context.

3.1 (Step 1) Select the model linking `normd15n` and `manure_level`

We first fit a model linking `normd15n` and `manure_level`. We have the same `modern` data as Styring et al. (2017) and the same model, a hierarchical normal linear model, denoted `HM`, regressing `normd15n` on `log(rainfall)`, with a random effect on the intercept due to `site`, a random effect due to `manure_level` within site, and a variance offset for wheat over barley. In R formula notation, (using the `nlme` package (Pinheiro et al., 2019)):

```
> HMmodel <- nlme::lme( normd15n ~ log_rainfall + manure_level,
+                   random=~1|site/manure_level,
+                   weights=varIdent(form=~1|category),
+                   data=mod.dat, method='REML' )
```

The variable-dependencies in this model were chosen using physical considerations, deviance tests, goodness-of-fit plots and predictive testing as described in Section 3.2.1 and Appendix B of the statistical supplement of Styring et al. (2017). We include our own calculations leading to the same model selected by Styring et al. (2017) in our accompanying scripts. We fit this model to the modern data and estimate model parameters.

3.2 (Step 2) Impute the missing `manure_level` values

Single imputation is carried out as described in Section 3.2.2 of the statistical supplement to Styring et al. (2017). When we fix a value for an unknown manure level we fix the fitted `normd15n`-value for that observation in the `HM` module. Imputed manuring level values `manure_level.imputed` are chosen to minimise the sum of squared differences between fitted and observed values for `normd15n`.

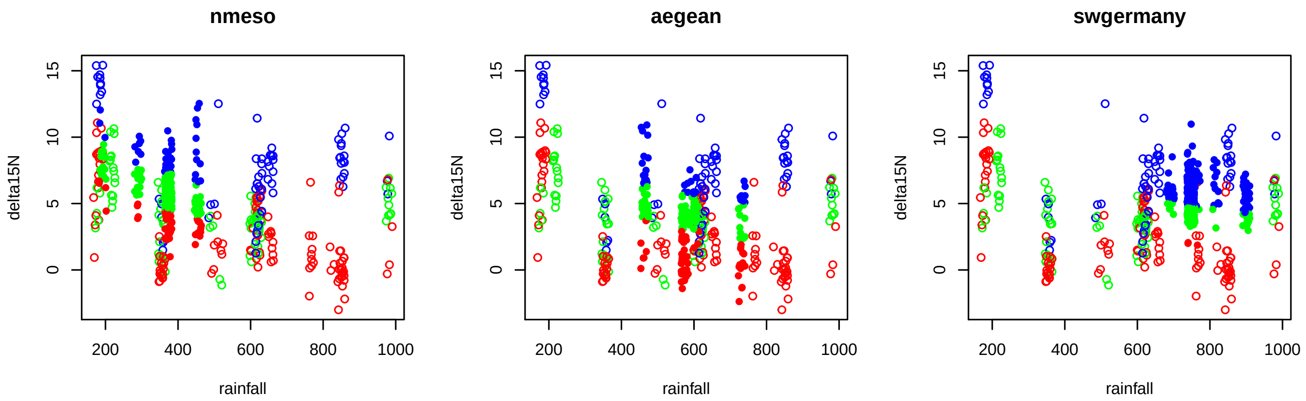

Figure 4: Observed `normd15n`-values colored by imputed manure level for the three archaeological datasets. Each panel shows the relation between `normd15n` and log(rainfall); each point corresponds to a single cereal grain-sample. modern(Archaeological) data plotted with open(filled) circles. Colors correspond to manuring level (red=low, green=medium, blue=high). For the modern data the manuring levels are known, for the Archaeological data, these are the `manure_level.imputed`-values.

3.3 (Step 3) Test for an association between the imputed manure level and site size

We can now estimate the effect of site `size` on `manure_level.imputed` in the archaeological data. We use a *Proportional Odds model* (denoted as `PO` hereafter) (McCullagh, 1980) with 3-level ordinal response `manure_level.imputed` and `size` as a potential explanatory variable. Proportional Odds models are GLM-like models widely used for modelling the dependence of an ordinal response on categorical and continuous covariates. See e.g. Agresti (2002) for more details of the model. `HM` and `PO` are submodels or *modules* of the overall model.

We carry out model selection on the covariates of the archaelogical data in order to choose the model for `PO`. Physical considerations must play a role as there are many models, and we need to avoid data-dredging. We compare models using information criteria (BIC and AIC) and deviance tests. In (Styring et al., 2017) model selection was carried out on the `nmeso` dataset. Effects due to `date` and random effects due to `phase` must be investigated on physical grounds. However these effects were not supported by the data in the `nmeso` case. The final selected model has a fixed effect due to `size`, a random effect on the `site` id, and a logit link function. In R formula notation (using the ordinal package (Christensen, 2010)),

```
> PO_nmeso = ordinal::clmm( manure_level.imputed ~ scale(size) + (1|site),
+                            link = "logit",
+                            data = agricurb_data %>%
+                              dplyr::filter( dataset=="nmeso" ) %>%
+                              dplyr::mutate( site=as.factor(site) ) )
> coefficients(summary(PO_nmeso))

              Estimate Std. Error   z value      Pr(>|z|)
low|medium  -1.5326947  0.4107875 -3.731113 0.00019063570
medium|high  1.6673236  0.4150418  4.017242 0.00005888314
scale(size) -0.4636204  0.1715456 -2.702607 0.00687980288
```

The first two rows in the output table give the `PO` module intercepts separating the levels of `manure_level.imputed`, and fit the simple overall proportion of `manure_level.imputed`-values in each level. The estimated coefficient for `size`, -0.4636, is negative and significant (p-value=0.0069). This means that the probability for a higher manure level tends to decrease as site-size increases, in agreement with Styring et al. (2017), and supporting their main finding of agricultural extensification in the `nmeso` dataset.

We applied the same reasoning to our two new datasets. For the `aegean` dataset, we arrive at a similar model, with the difference that `size` is included on a logarithmic scale for best fit.

```
> PO_aegean = ordinal::clmm( manure_level.imputed ~ scale(log_size) + (1|site),
+                             link = "logit",
+                             data = agricurb_data %>%
+                               dplyr::filter( dataset=="aegean" ) %>%
+                               dplyr::mutate( site=as.factor(site) ) %>%
+                               dplyr::mutate( phase=as.factor(phase) ) )
> coefficients(summary(PO_aegean))

                  Estimate Std. Error   z value   Pr(>|z|)
low|medium      -0.9288111  0.5528567 -1.680021 0.09295315
medium|high      1.6158724  0.5673630  2.848040 0.00439894
scale(log_size) -0.5076650  0.2335632 -2.173566 0.02973775
```

The estimated coefficient for `size`, -0.5077, is negative and significant (p-value=0.0297), providing evidence that manure level outcome decreases with increasing site size at this site as well.

We found no effect due to `date` in the `aegean` data. However, a model with random effects on `site` and `phase` but not `size` performs as well as `PO_aegean` given above. We have then to choose between `size` and `phase`. The experimental design in the `aegean` data makes it hard to disentangle the two variables. This is due to colinearity between `size` and `site:phase`: there are few observations at each level defined by `site:phase` and they share the same value of `size` within each level. Any change in the linear predictor associated with variation of the variable `size` can be reproduced using a linear combination of the dummy variables associated with `site:phase`. We kept `size` and dropped `phase` based on two considerations: 1) the results from the `nmeso` data, where the experimental design allows us to separate the effects of the two factors, and the high-level similarities between

agricultural practices in the two cultures; and 2) the higher dimension of the random effects for the categorical variable `phase` make it possible for any effect attributable to the one dimensional continuous variable `size` to be represented as an effect due to `phase`, but not in general vice-versa. We accept the more parsimonious explanation.

In the `swgermany` data, we will see that `size` is not a significant explanatory variable. If it is included, the associated coefficient is positive, but not significant. The `swgermany` data also show qualitatively different covariate dependence to the other datasets. The model selected using information criteria and deviance tests includes random effects on `site` ID, but does not include `size`. We preserve log-size in the model in later analysis as this effect is of interest. However we can expect the associated parameter to be close to zero.

```
> PO_swgermany = ordinal::clmm( manure_level.imputed ~ scale(log_size) + (1|site),
+                             link = "logit",
+                             data = agricurb_data %>%
+                               dplyr::filter( dataset=="swgermany" ) %>%
+                               dplyr::mutate( site=as.factor(site) ) )
> coefficients(summary(PO_swgermany))

                  Estimate Std. Error    z value               Pr(>|z|)
low|medium      -5.4747248  0.7170897 -7.6346445 0.00000000000002264445
medium|high     -1.1441110  0.4281816 -2.6720230 0.00753954827467872256
scale(log_size)  0.2503616  0.3679126  0.6804919 0.49619307069162338353
```

The estimated coefficient associated with `size` in the `swgermany` data, 0.2504, is positive but not significant (p-value=0.4962).

3.4 Conclusions from Single Imputation

The effect of site `size` on `manure_level.imputed` in the `nmeso` and `aegean` datasets is significant at level 0.05 (respectively, p=0.0069 and p=0.0297). This agrees with previous work on the `nmeso` data and supports the hypothesis of extensification in the new `aegean` data. The effect due to `size` in the `aegean` data is hard to separate from the effect due to `phase`. We report the test for the more parsimonious model. The effect in the `swgermany` data is not significant (p=0.5), so we find no support for extensification at the `swgermany` sites.

Single imputation conditions on a single set of `manure_level.imputed`-values, so it does not allow for uncertainty in imputed values. Our analysis above also ignored uncertainty in rainfall values for Archaeological contexts. However, we will see that the results from our most careful analysis (SMI, summarised in section 5.3) are in good agreement, on all three datasets, with the conclusions of the simple analysis given in this section.

4 Bayesian Multiple Imputation (BMI)

In this section we give a Bayesian analysis of the effect of site size on manuring levels in the archaeological data. We account for essentially all sources of uncertainty in this analysis. In section 4.2, we report that model misspecification undermines a conventional *full* Bayesian approach to this particular problem. In section 4.3 we use Bayesian Multiple Imputation (BMI), following the Styring et al. (2017) analysis of the `nmeso` data. We expand the model used by those authors by including a possible effect due to `date` in the `PO` module. The effect due to `date` was not found significant in the single imputation analysis. We nevertheless include it in the Bayesian analysis in order to make that analysis self-contained, and because it is has been raised as a possible confounder. BMI is relatively robust to misspecification (see Lunn et al. (2009); Plummer (2015) where it is referred to as a “cut model”). Thousands of sets of `manure_level.imputed` values are sampled according to their posterior distribution in the `HM` module. Each set of `manure_level.imputed` values determines a posterior distribution for the size-effect. These distributions are averaged to get a posterior for the effect of interest which allows for uncertainty in the unknown manure levels (and `rainfall`).

Our full R-implementation of the methods used in this document is available via the R package `agricurbayes`. Supplementary scripts replicate the results and figures in this section using functions available in that package.

4.1 Common notation

We begin with the `HM` module introduced in section 3. Let Y_{arc}, Y_{mod} be the `normd15n`-values in the archaeological and modern data, respectively. Let M_{arc} and R_{arc} respectively denote the unknown true manure and rainfall levels

for the archaeological data. Here M_{arc} plays the same role as `manure_level.imputed` in the single imputation above. Let $\beta = (\beta_1, \ldots, \beta_4)$ be the four fixed effects in the HM module associated with the intercept, R_{arc} and M_{arc} (three levels, so two offsets for medium and high levels relative to baseline which is low-level manuring). The sets of site-level ID's are

$$S_M = \{s; s \text{ is an ID for a site in the modern dataset}\},$$

and

$$S_A = \{s; s \text{ is an ID for a site in the archaeological dataset under analysis}\},$$

and let $\zeta = (\zeta_s)_{s\in\{S_A \cup S_M\}}$ be the mean-zero normal random effects for `site`-levels for the modern and archaeological site, with variance σ^2_ζ. We make a separate analysis for each archaeological dataset, so the site ID's in S_A, the dimension of ζ, and σ^2_ζ vary from one analysis to another. Finally, let σ^2 be the variance of the `normd15n` response given the value of its linear predictor. Denote by $P(Y_{mod} \mid \beta, \zeta)$ the observation model density for the modern data in the HM module, where manuring levels are known, and by $P(Y_{arc} \mid \beta, \zeta, M_{arc})$ the same for the archaeological data, where they are unknown.

For the Proportional Odds module PO, let γ denote the effect due to `size` on manure level M_{arc} in the archaeological data under analysis. Let τ denote the corresponding effect due to `date`. Let $\alpha = (\alpha_1, \alpha_2)$ be the intercepts for our two-level ordinal PO-response M_{arc}. Let $\xi = (\xi_s)_{s\in S_A}$ be mean-zero normal random effects due to `site` on manure-level (only relevant for archaeological sites), with variance σ^2_ξ. Let $P(M_{arc} \mid \alpha, \gamma, \tau, \xi)$ be the (proportional-odds) probability mass function for the missing manure-levels in the archaeological data.

Denote by $\phi = (\alpha, \tau, \xi, \sigma_\xi)$ all the variables of the PO module except γ. Denote by $\theta = (\beta, \zeta, \sigma, \sigma_\zeta, R_{arc})$ all the variables of the HM module except M_{arc}.

Our parameter of interest is γ, the effect due to size on manure level, which is linked to the hypothesis of extensification.

4.2 Remarks on full Bayesian Inference and priors

In the following we take flat prior distributions, $P(\beta) \propto 1$, $P(\alpha, \gamma, \tau) \propto 1$, subject to conservative upper and lower bounds $(\alpha, \gamma, \tau) \in [-5, 5]^3$ on the PO module parameters. These are justified below.

The prior for the variance of the random effects in both models is an inverse gamma, $\sigma_\zeta, \sigma_\xi \sim \text{InvGamma}(\alpha = 2, \beta = 1)$. This prior concentrates most of its probability (around 70%) in values smaller than 1 and gives enough room for larger values.

The prior for rainfall $P(R_{arc})$ is $R_{arc} \sim U(\texttt{min\_rainfall}, \texttt{max\_rainfall})$ as these bounds are fairly tight. The joint posterior distribution conditional on all observed data is

$$\begin{aligned} P(\alpha, \gamma, \tau, \xi, M_{arc}, \beta, \zeta, M_{arc}, R_{arc} \mid Y_{mod}, Y_{arc}) \propto & P(Y_{mod} \mid \beta, \zeta)\ P(Y_{arc} \mid \beta, \zeta, M_{arc}, R_{arc}) \cdot \\ & P(R_{arc})\ P(\zeta \mid \sigma_\zeta)\ P(\beta, \sigma_\zeta) \cdot \\ & P(M_{arc} \mid \alpha, \gamma, \tau, \xi) P(\xi \mid \sigma_\xi)\ P(\alpha, \gamma, \tau, \sigma_\xi). \end{aligned} \tag{1}$$

Explicit expressions for these densities and probability mass functions are given in Styring et al. (2017).

The parameters of the HM module are well-informed by the data, and any reasonable prior leads to the same conclusions, so we make no careful prior elicitation for that module. However, (Styring et al., 2017) point out that, without a proper prior for the parameters of the PO module, the posterior is improper (the normalising constant is infinite).

This is related to the problem of linear separability in multinomial logistic regression (Albert and Anderson, 1984), where a maximum likelihood estimate need not exist, and is compounded by our use of data-augmentation in the PO module, where M_{arc} is missing data. Our conservative bounds make the prior proper and remove this problem.

We have then to justify our choice of bounds. Firstly, values of α and γ outside $[-5, 5]$ give models in which very little variation in manure-level is allowed at a site. It may be argued that this is not completely un-physical. However, values as large as $\pm 4, \pm 5$ are already strongly informative. Also, the outcome of our test is not sensitive to the choice of bounds: we measure evidence for the hypothesis that $\gamma < 0$; the absolute value of γ is not of interest. We have experimented with more extreme bounds and find that the evidence for $\gamma < 0$ in the `nmeso` and `aegean` data only increases. However, although the dependence of the evidence on widening bounds is understood, accurate MCMC estimation of the evidence becomes impractical for significantly wider bounds. Further exploration was in any case uninteresting for the reasons given above.

However, there is a further problem. There is model misspecification in the PO module, at least for the `nmeso` and `aegean` data. This is discussed in Styring et al. (2017) and motivated their use of misspecification-robust BMI. The

most straightforward evidence for misspecification is found in the SMI analysis below, where the control parameter η is a summative measure of misspecification. Our estimated values show that misspecification is present. The model elaborations we explored to identify and correct misspecification did not remove the problem.

If we naively apply full Bayesian inference then we find the posterior for γ puts all its mass close to the lower bound $\gamma \simeq -5$ and follows the lower bound as it is moved to lower values. This cannot be accepted as evidence that γ is negative as the model is misspecified. Repeating the analysis on synthetic data with the same covariates as the real data, where there is no model misspecification, gives posteriors for γ which cover the fixed synthetic true value, with tails which go rapidly to zero, so that conservative upper and lower limits on γ play no practical role in the analysis. This qualitatively different behaviour for synthetic data is further evidence for misspecification.

Continuing the reasoning of (Styring et al., 2017), we could impose strongly informative prior distributions for parameters of the `PO` module or M_{arc} itself.

However, we have no concrete prior information justifying informative priors on the PO-module parameters, or restrictions on M_{arc}. Styring et al. (2017) refer to the literature on model misspecification in evidence combination. This treats misspecification by replacing full Bayesian inference with BMI, also known as "cut models". This is the approach we now apply.

4.3 Bayesian Multiple Imputation analysis

The analysis presented here follows the BMI-analysis for the `nmeso` data in Styring et al. (2017). We make some slight revisions and extend the analysis to the `aegean` and `swgermany` data. The analysis is applied to one dataset at a time (with the same modern calibration data used throughout).

(Step 1) This is the imputation step. We sample the posterior for M_{arc} in the `HM` module. In the full Bayesian analysis the `PO` module acts as a prior on M_{arc}. Here we cut that feedback and take a flat prior, $P(M_{arc})$. Parameter priors, collectively $P(\theta)$, are otherwise unchanged. The imputation posterior is

$$P(M_{arc}, \theta \mid Y_{mod}, Y_{arc}) \propto P(Y_{mod}, Y_{arc} \mid \theta, M_{arc})\; P(\theta)P(M_{arc}). \tag{2}$$

In terms of the low-level variables,

$$P(Y_{mod}, Y_{arc} \mid \theta, M_{arc}) = P(Y_{mod} \mid \beta, \zeta)\; P(Y_{arc} \mid \beta, \zeta, M_{arc}, R_{arc}),$$

as before (first line of eq. (1)).

(Step 2) We now treat M_{arc} as data in the `PO` module. All `PO` priors, collectively $P(\phi)$, are unchanged. The `PO` posterior conditioned on M_{arc} is

$$P(\gamma, \phi \mid M_{arc}) = \frac{P(M_{arc} \mid \phi, \gamma)\; P(\phi)}{D(M_{arc})}, \tag{3}$$

where $D(M_{arc})$ is an intractable normalising constant. In terms of the low-level variables,

$$P(M_{arc} \mid \phi, \gamma) = P(M_{arc} \mid \alpha, \gamma, \tau, \xi),$$

as in eq. (1).

Many of the model elements are unchanged from the deprecated full Bayes analysis. However, we now carry out MCMC targeting

$$P_{BMI}(\gamma, \phi, M_{arc}, \theta \mid Y_{mod}, Y_{arc}) \equiv P(\gamma, \phi \mid M_{arc})P(M_{arc}, \theta \mid Y_{mod}, Y_{arc}), \tag{4}$$

the revised BMI-posterior for γ.

The dependence on $D(M_{arc})$ may look awkward, but is straightforward to handle using Monte Carlo methods. The M_{arc} distribution is determined by the posterior in eq. (2), and then for any fixed M_{arc}, we can sample from $P(\gamma, \phi \mid M_{arc})$ in eq. (3) using standard MCMC methods. This sampling scheme follows Plummer (2015). The MCMC algorithm is outlined in Styring et al. (2017). The `agricurbayes` package contains our independent implementation set out in detail in the supplement to Carmona and Nicholls (2020). fig. 5 shows the posterior distribution of γ under P_{BMI}.

We measure evidence in favour of extensification (manure level outcome decreases with increasing site size) using the posterior probability that the effect of size, γ, is negative.

$$P(\gamma < 0 \mid Y_{mod}, Y_{arc}) = \int_{-\inf}^{0} P_{BMI}(\gamma \mid Y_{mod}, Y_{arc})\, d\gamma \tag{5}$$

This probability is estimated using output from our MCMC algorithm targeting P_{BMI} in eq. (4).

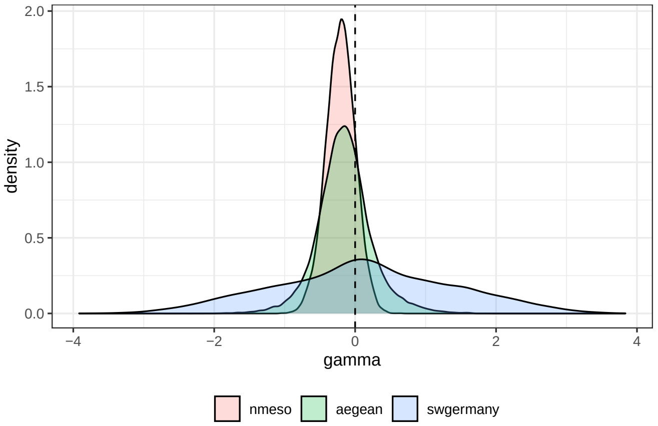

Figure 5: Posterior distribution of γ, the effect due to size on manuring levels in the archaeological data.

```
> # Posterior probability of negative gamma #
> p_gamma_leq_0 <- apply( mcmc_gamma < 0, 2, mean )
> p_gamma_leq_0

    nmeso    aegean swgermany
0.8421000 0.6926333 0.4554000
```

The posterior probability for $\gamma < 0$ in the `nmeso` data is 0.8421. The corresponding posterior probabilities for the other datasets may be read off from the output table. Our prior for γ puts equal weight on the two hypotheses $\gamma < 0$ and $\gamma \geq 0$ so the Bayes Factor (Kass and Raftery, 1995) $B_{\gamma<0}$ comparing these two hypotheses is

$$B_{\gamma<0}^{(BMI)} = \frac{P_{BMI}(\gamma < 0 \mid Y_{mod}, Y_{arc})}{P_{BMI}(\gamma \geq 0 \mid Y_{mod}, Y_{arc})}. \tag{6}$$

```
> # Bayes factor for negative gamma #
> BF_gamma_leq_0 = p_gamma_leq_0 / (1-p_gamma_leq_0)
> BF_gamma_leq_0

    nmeso    aegean swgermany
5.3331222 2.2534432 0.8362101
```

The `nmeso` and `aegean` datasets give Bayes factors equal to 5.3331 and 2.2534 respectively, evidence for $\gamma < 0$. This is in line with the findings in Styring et al. (2017) and section 3. These Bayes factors are likely to underestimate the strength of the evidence due to dilution from noise in the imputation. The `swgermany` data gave a Bayes factor equal to 0.8362, close to 1, and indicating no evidence for a negative γ. The posterior distribution is centred around zero so the effect is not significant here. This is all in line with our findings in section 3.

4.4 Conclusions from Bayesian Multiple Imputation

We find moderate evidence for a negative effect of site `size` on manure level in the `nmeso` and `aegean` datasets (respectively, $B_{\gamma<0}^{(BMI)}$ =5.33 and $B_{\gamma<0}^{(BMI)}$ =2.25). This agrees with previous work on the `nmeso` data and does not contradict the hypothesis of extensification in the new `aegean` data. We find no evidence for an effect in the `swgermany` data ($B_{\gamma<0}^{(BMI)}$ =0.8362) so no support for extensification at the `swgermany` sites. These measures allow for all major sources of uncertainty (including now any possible confounding due to `date`). They are conservative due to the effect of dilution in BMI.

5 Semi-Modular Inference

The strength of the evidence for $\gamma < 0$, the effect of `size` on M_{arc}, measured using BMI in the `nmeso` and `aegean` datasets in section 4 is real but not overwhelming. Recent work on evidence combination, in particular *Semi-Modular Inference (SMI)* (Carmona and Nicholls, 2020), adjusts for misspecification without dilution.

5.1 Outline of SMI

Semi-Modular Inference (SMI) combines BMI (Liu et al., 2009; Lunn et al., 2009; Plummer, 2015) and the power-posterior (Bissiri et al., 2016; Holmes and Walker, 2017; Grünwald and van Ommen, 2017), two inferential procedures treating model misspecification in Bayesian inference. The contribution each module (here `HM` and `PO`) makes to the inference is controlled by a parameter η so that misspecified modules can be down-weighted. In a misspecified setting this may give more accurate imputation and hence better downstream inference than either of fully Bayesian inference or BMI. It will not in general do worse. This is because SMI defines a continuum of inference procedures, indexed by η, interpolating between BMI (at $\eta = 0$) and fully Bayesian inference (at $\eta = 1$). It includes these methods as special cases, and selects the procedure with best predictive performance.

In our context, we impute M_{arc} using an η-*tempered* version of the full model, which reduces (but does not eliminate) the influence of the `PO` module by raising its likelihood to a power $\eta \in [0, 1]$. This setup is used in the imputation of M_{arc}. The second stage of the analysis is the same as step 2 of BMI. In the notation of section 4 the SMI posterior for our model is

$$P_\eta(\gamma, \phi, \tilde{\gamma}, \tilde{\phi}, M_{arc}, \theta \mid Y_{mod}, Y_{arc}) = P(\gamma, \phi \mid M_{arc})\ P_\eta(\tilde{\gamma}, \tilde{\phi}, M_{arc}, \theta \mid Y_{mod}, Y_{arc}). \tag{7}$$

Here $\tilde{\gamma}$ and $\tilde{\phi}$ are parameters used only at the imputation stage, $P(\gamma, \phi \mid M_{arc})$ is unchanged from eq. (3) and

$$\begin{aligned} P_\eta(\tilde{\gamma}, \tilde{\phi}, M_{arc}, \theta \mid Y_{mod}, Y_{arc}) \propto & P(Y_{mod} \mid \beta, \zeta)\ P(Y_{arc} \mid \beta, \zeta, M_{arc}, R_{arc}) \cdot \\ & P(R_{arc})\ P(\zeta \mid \sigma_\zeta)\ P(\beta, \sigma_\zeta) \cdot \\ & P(M_{arc} \mid \alpha, \gamma, \tau, \xi)^\eta P(\xi \mid \sigma_\xi)\ P(\alpha, \gamma, \tau, \sigma_\xi). \end{aligned} \tag{8}$$

Note the similarity between eq. (4) (BMI-posterior) and eq. (7) (SMI posterior). The two-stage inference set out in Plummer (2015) remains, but the BMI imputation posterior in eq. (1) is replaced in the SMI imputation posterior (eq. (8)) by something very like the full posterior in eq. (1). The change is that the `PO` module likelihood in eq. (8) is raised to a power η to reduce its influence. The SMI-imputation step uses a power posterior.

We have finally to choose a value for η. Following Carmona and Nicholls (2020), we define the optimal η as the value $\eta = \eta^*$ say, which maximises the Expected Log Predictive Density or *ELPD* (Vehtari et al., 2017) for the `normd15n`-data. This is a natural performance measure, accounting for uncertainty in unknown parameters. It is equivalent to carrying out Leave-One-Out Cross-Validation (LOOCV) on held out `normd15n` responses and selecting the η-value that minimises the mean square error for prediction. It can be computed from the MCMC output. Further details of SMI are given in Carmona and Nicholls (2020).

We use the priors given in section 4.2 and the observations models `HM` and `PO` given in section 3.

5.2 Results from SMI

We use MCMC to sample P_η in a two-step procedure similar to that outlined for P_{BMI}.

In fig. 6 we show the estimated *ELPD* as a function of $\eta \in [0, 1]$.

The optimal values for η, denoted η^*, are approximately 0.8 for `nmeso`, 0.55 for `aegean`, and 1 for `swgermany`. The fact that $\eta^* < 1$ in the `nmeso` and `aegean` data is evidence for misspecification. There is no evidence for misspecification in the `swgermany` data where the optimal imputation scheme is full Bayes itself.

We plot in fig. 7 credible intervals for the SMI-posterior of γ, the effect of `size` on manure level M_{arc}, as a function of the control parameter η.

The plots show the posterior mean (solid line) and the 95% and 50% credible intervals (shades) at each η-value (ie each vertical slice). Panels correspond to datasets `nmeso` (left), `aegean` (centre), and `swgermany` (right). The vertical lines give the optimal value of η in each dataset. Our selected SMI posterior is the posterior given by the credible intervals at this line and is shown in fig. 8 below. Referring to fig. 7, the `nmeso` and `aegean` datasets (left and centre) support similar negative values for the size effect γ at all values of the control parameter η, whereas the `swgermany`-posterior is close-to or slightly above zero.

The posterior credible interval for γ determined by BMI in section 4.3 (fig. 5), and Styring et al. (2017) supplement Figure 7. (left) page 16, can be read off at $\eta = 0$ in the leftmost `nmeso` plot. Credible intervals shrink towards zero as $\eta \to 0$ as imputation noise rises. The posterior diverges as η approaches $\eta = 1$ and we move towards fully Bayesian inference. This is a consequence of the combined effects of misspecification, linear separability and the conservative bounds used in our non-informative priors, as discussed in section 4.2.

fig. 8 shows the posterior distribution of γ at $\eta = \eta^*$ for each dataset. For the `nmeso` and `aegean` datasets, the posterior distribution puts little weight on $\gamma > 0$, evidence of a negative effect of `size` on estimated M_{arc}-values.

The evidence in favor of the agricultural extensification hypothesis is measured by the posterior probability that the effect γ is negative, $P_{\eta^*}(\gamma < 0 \mid Y_{mod}, Y_{arc})$.

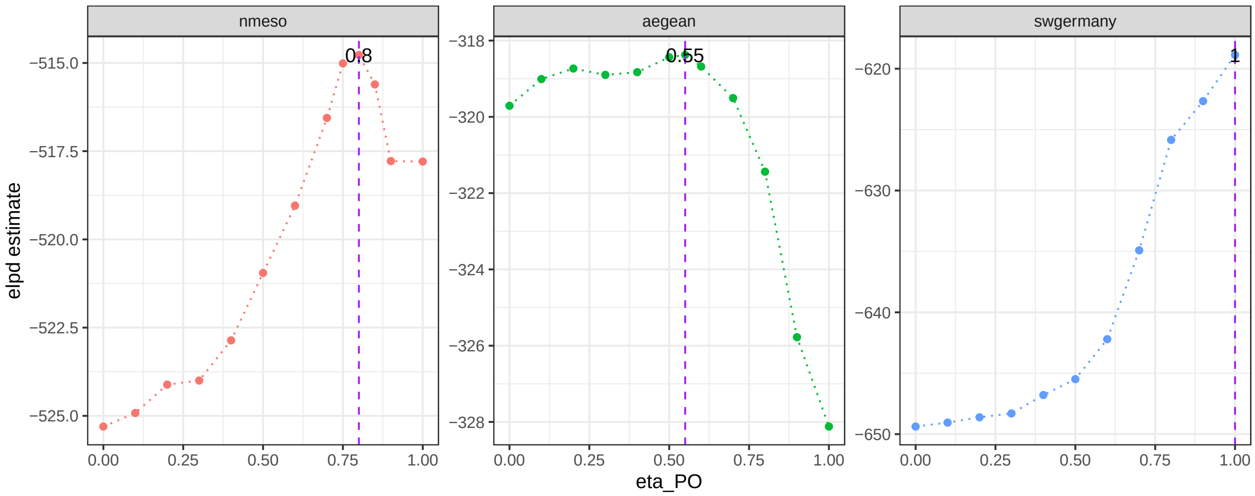

Figure 6: Estimated predictive performance measured by ELPD (y-axis) as a function of the control parameter η (x-axis) under SMI for each of the three datasets (see panel titles). Vertical lines give the optimal values $\eta = \eta^*$ of the control parameter for each dataset.

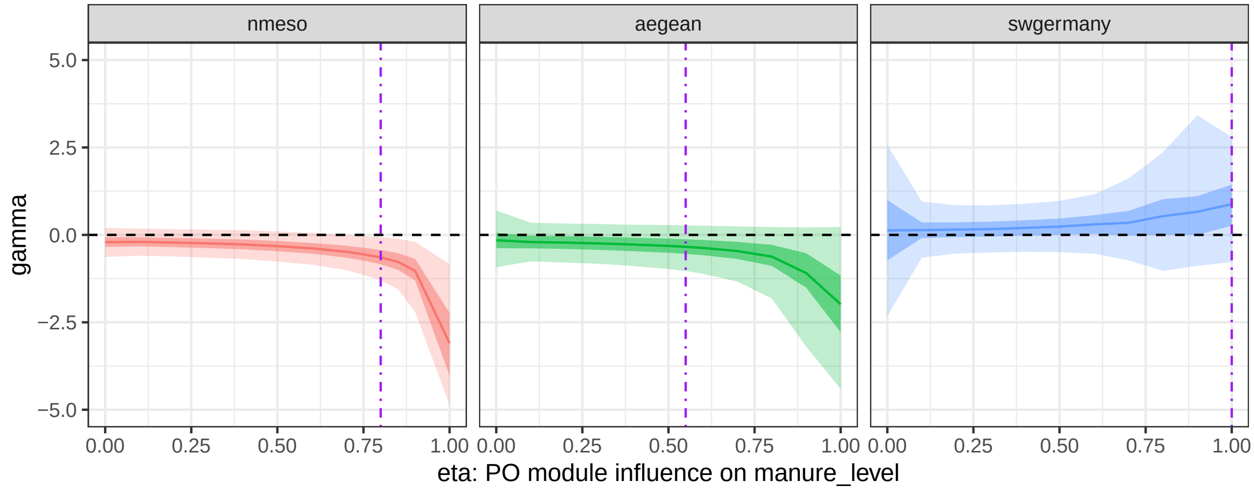

Figure 7: Posterior mean and credible intervals of γ under Semi-Modular Inference. The y-axis correspond to the value of γ, while the x-axis corresponds to values of the degree of influence $\eta \in [0, 1]$.

```
> # Posterior probability of negative gamma #
> p_gamma_leq_0_smi <- apply( mcmc_best_smi_gamma < 0, 2, mean )
> p_gamma_leq_0_smi

    nmeso    aegean swgermany
0.9849333 0.8615000 0.1554333
```

The posterior probabilities for $\gamma < 0$ is higher in `nmeso` and `agean` than we saw in BMI, section 4 as dilution is reduced. Some evidence for $\gamma > 0$ in `swgermany` now appears.

The corresponding Bayes Factors for the hypothesis $\gamma < 0$ are given by

$$B^{(\eta^*)}_{\gamma<0} = \frac{P_{\eta^*}(\gamma < 0 \mid Y_{mod}, Y_{arc})}{1 - P_{\eta^*}(\gamma < 0 \mid Y_{mod}, Y_{arc})}$$

```
> # Bayes factor for negative gamma #
> BF_gamma_leq_0_smi = p_gamma_leq_0_smi / (1-p_gamma_leq_0_smi)
> BF_gamma_leq_0_smi
```

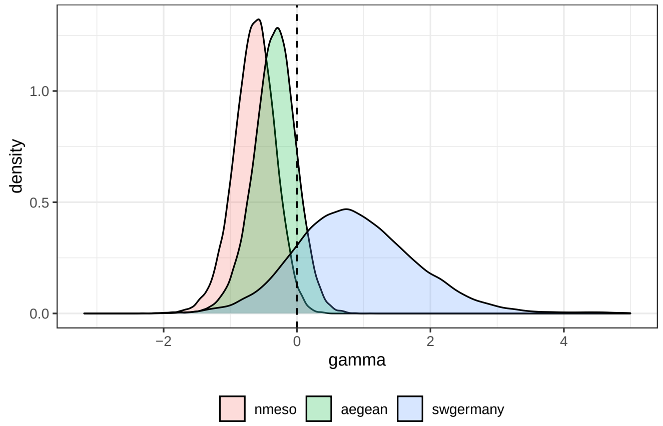

Figure 8: Posterior distribution of γ under SMI for the optimal degree of influence, η^*.

```
     nmeso     aegean  swgermany
65.3716814  6.2202166  0.1840392
```

These Bayes factors (65 and 6) give strong support for $\gamma < 0$ in the `nmeso` and substantial support in the `aegean` data. The Bayes factor for $\gamma < 0$ in the `swgermany` data (0.184) gives substantial evidence for $\gamma > 0$.

5.3 Conclusions from SMI

We find evidence for a negative effect of site `size` on imputed manure level M_{arc} in the `nmeso` and `aegean` datasets (respectively, $B_{\gamma<0}^{(\eta^*)}$ =65.3717 and $B_{\gamma<0}^{(\eta^*)}$ =6.2202). This improves on the conservative BMI estimate for the `nmeso` data (taking into account any possible effect due to `date`) and provides clear evidence for the hypothesis of extensification in the new `aegean` data.

There is some evidence for a positive effect in the `swgermany` data ($B_{\gamma<0}^{(\eta^*)}$ =0.184, or in other words a Bayes factor of about 5.4 for the opposite hypothesis, that γ is positive). The conclusion is less clear in this case, and not only for the reason that the evidence is weaker. We entered the analysis aiming to measure evidence for $\gamma < 0$ and so there is no question, for the `nmeso` and `aegean` analyses, of our changing the hypothesis on seeing the data. In claiming evidence for $\gamma > 0$ in the `swgermany` analysis we would be changing our hypothesis after seeing the data. This undermines the analysis (see discussion in Cox (2006)) and, taken with the real but not overwhelming strength of the evidence (Bayes Factor 5.4), we would be inclined to leave this as undecided.

Finally, we visualise the size effect. In fig. 9 we show the relation between size and manuring level estimated at $\eta = \eta^*$. The pair of plots in each row share a dataset, `nmeso` (top), `aegean` (middle), and `swgermany` (bottom). Plots in the left column show, for each missing manure level in M_{arc}, the posterior probability the missing level takes the value *low* as a function of `size`. The right column shows the posterior probability for the missing manure level to take either of the values *low* or *medium*. Tighter credible intervals (shaded area) indicate a clearer relation between estimated M_{arc} and `size`. The negative effect of γ is evident for `nmeso` and `aegean`: the posterior probability for lower manure levels grows as `size` increases. For `swgermany` the effect is positive.

6 Overall conclusions

We reported results from three analyses of our data. Single imputation gives a simple introduction to the models and inference problem and gave an easy-to-understand check on the more sophisticated analyses which followed. Bayesian Multiple Imputation is a familiar method, allowed direct comparison with Styring et al. (2017), and has much in common with Semi-Modular Inference.

All three analyses, single imputation (section 3), BMI (section 4) and SMI (section 5) give consistent evidence for the hypothesis of extensification in the `nmeso` and `aegean` data. In the main paper we report the SMI measures reported in section 5.3,as this is our favored analysis. It improves on single imputation as it allows for all major sources of uncertainty and improves on BMI as it greatly reduces the dilution effect we saw in BMI (see in particular our comments on fig. 7).

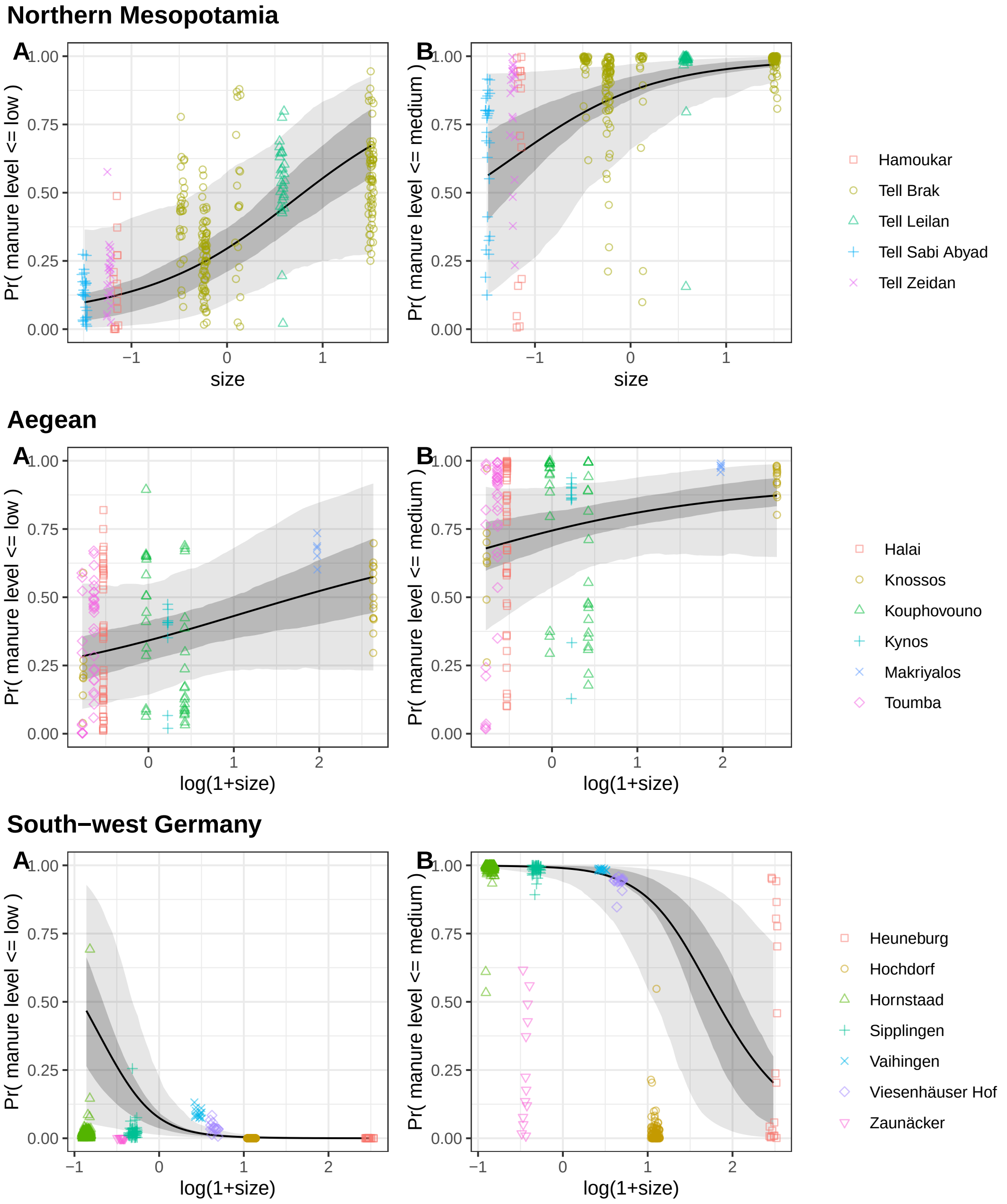

Figure 9: The posterior probability (y-axis) that a given archaeological cereal grain sample has a manuring level equal m or lower is plotted against site size (x-axis). (a) Manuring level m=low. (b), Manuring level m=medium. Shaded areas give 50% and 90% credible intervals for the probability. Each row corresponds to a dataset: `nmeso` (top), `aegean` (middle), and `swgermany` (bottom). Colors distinguish different sites.

References

Agresti, A. (2002). *Categorical Data Analysis*. Wiley Series in Probability and Statistics. John Wiley & Sons, Inc., Hoboken, NJ, USA.

Albert, A. and Anderson, J. A. (1984). On the Existence of Maximum Likelihood Estimates in Logistic Regression Models. *Biometrika*, 71(1):1.

Bissiri, P. G., Holmes, C. C., and Walker, S. G. (2016). A general framework for updating belief distributions. *Journal of the Royal Statistical Society: Series B (Statistical Methodology)*, 78(5):1103–1130.

Carmona, C. U. and Nicholls, G. K. (2020). Semi-Modular Inference: enhanced learning in multi-modular models by tempering the influence of components. In *Proceedings of the 23rd International Conference on Artificial Intelligence and Statistics, AISTATS 2020*.

Christensen, R. H. B. (2010). Ordinal—regression models for ordinal data.

Cox, D. R. (2006). *Principles of Statistical Inference*. Cambridge University Press.

Grünwald, P. and van Ommen, T. (2017). Inconsistency of Bayesian Inference for Misspecified Linear Models, and a Proposal for Repairing It. *Bayesian Analysis*, 12(4):1069–1103.

Holmes, C. C. and Walker, S. G. (2017). Assigning a value to a power likelihood in a general Bayesian model. *Biometrika*, 104(2):497–503.

Kass, R. E. and Raftery, A. E. (1995). Bayes Factors. *Journal of the American Statistical Association*, 90(430):773–795.

Knuiman, M. W., Divitini, M. L., Buzas, J. S., and Fitzgerald, P. E. (1998). Adjustment for Regression Dilution in Epidemiological Regression Analyses. *Annals of Epidemiology*, 8(1):56–63.

Liu, F., Bayarri, M. J., and Berger, J. O. (2009). Modularization in Bayesian analysis, with emphasis on analysis of computer models. *Bayesian Analysis*, 4(1):119–150.

Lunn, D., Best, N., Spiegelhalter, D., Graham, G., and Neuenschwander, B. (2009). Combining MCMC with 'sequential' PKPD modelling. *Journal of Pharmacokinetics and Pharmacodynamics*, 36(1):19–38.

McCullagh, P. (1980). Regression Models for Ordinal Data. *Journal of the Royal Statistical Society. Series B (Methodological)*, 42(2):109–142.

Pinheiro, J., Bates, D., DebRoy, S., Sarkar, D., and R Core Team (2019). nlme: Linear and Nonlinear Mixed Effects Models.

Plummer, M. (2015). Cuts in Bayesian graphical models. *Statistics and Computing*, 25(1):37–43.

Styring, A. K., Charles, M., Fantone, F., Hald, M. M., McMahon, A., Meadow, R. H., Nicholls, G. K., Patel, A. K., Pitre, M. C., Smith, A., Sołtysiak, A., Stein, G., Weber, J. A., Weiss, H., and Bogaard, A. (2017). Isotope evidence for agricultural extensification reveals how the world's first cities were fed. *Nature Plants*, 3(6).

Vehtari, A., Gelman, A., and Gabry, J. (2017). Practical Bayesian model evaluation using leave-one-out cross-validation and WAIC. *Statistics and Computing*, 27(5):1413–1432.

Appendix A. Pairwise relation of variables on modern and archaeological data

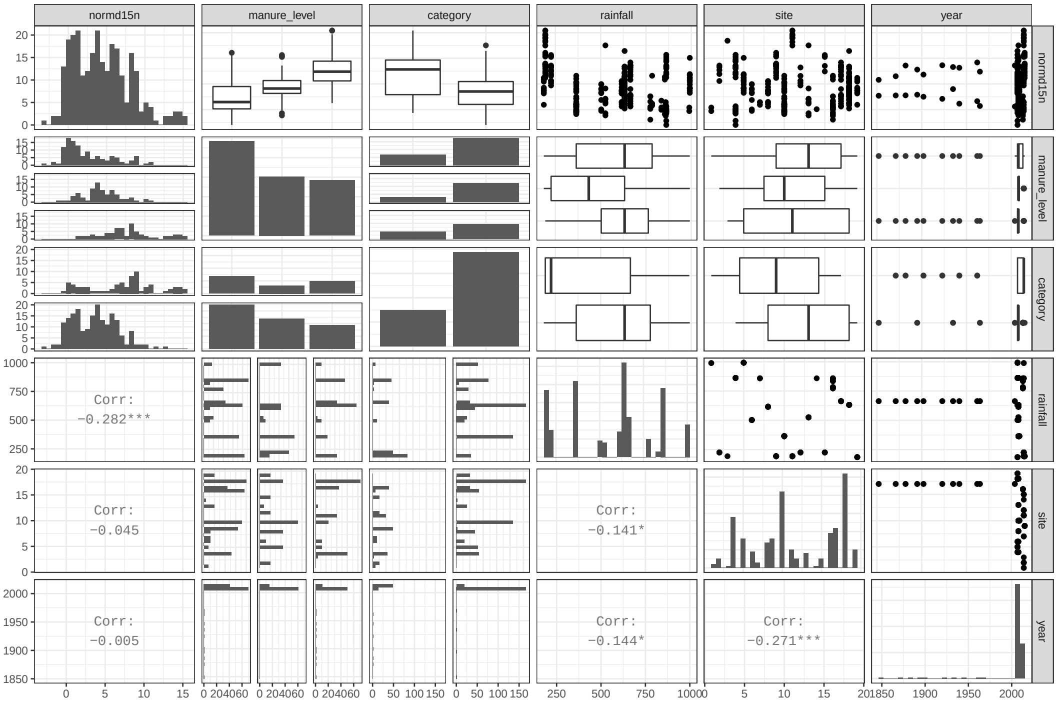

Figure 10: Pairwise relation between key variables in the modern data

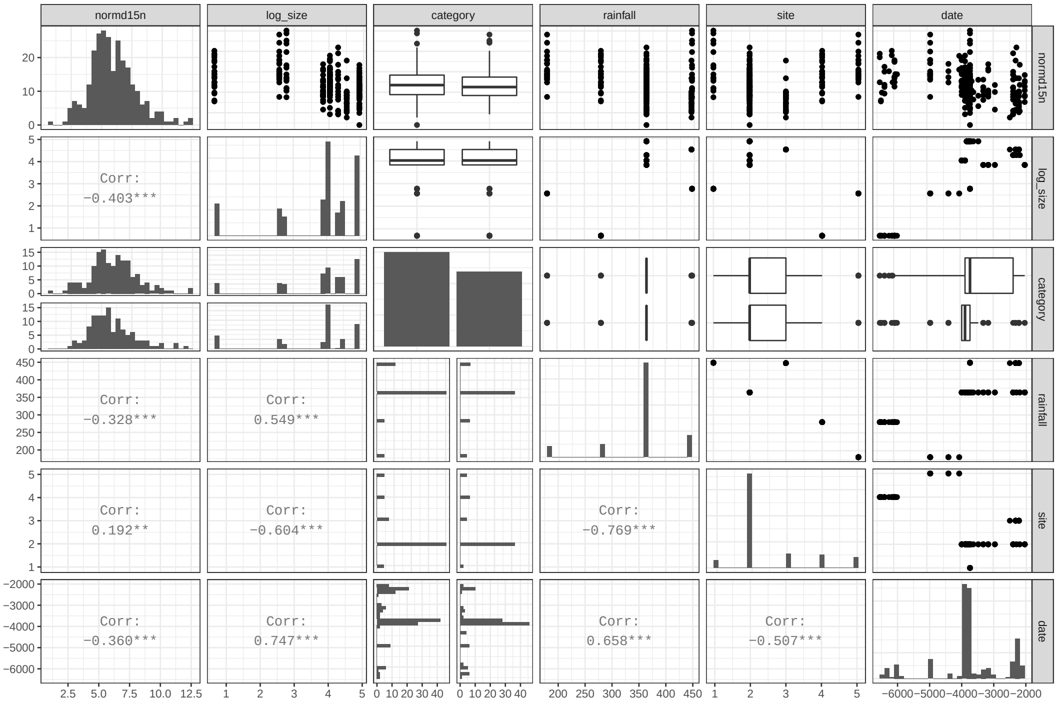

Figure 11: Pairwise relation between key variables in the archaeological data from Northern Mesopotamia (`nmeso`)

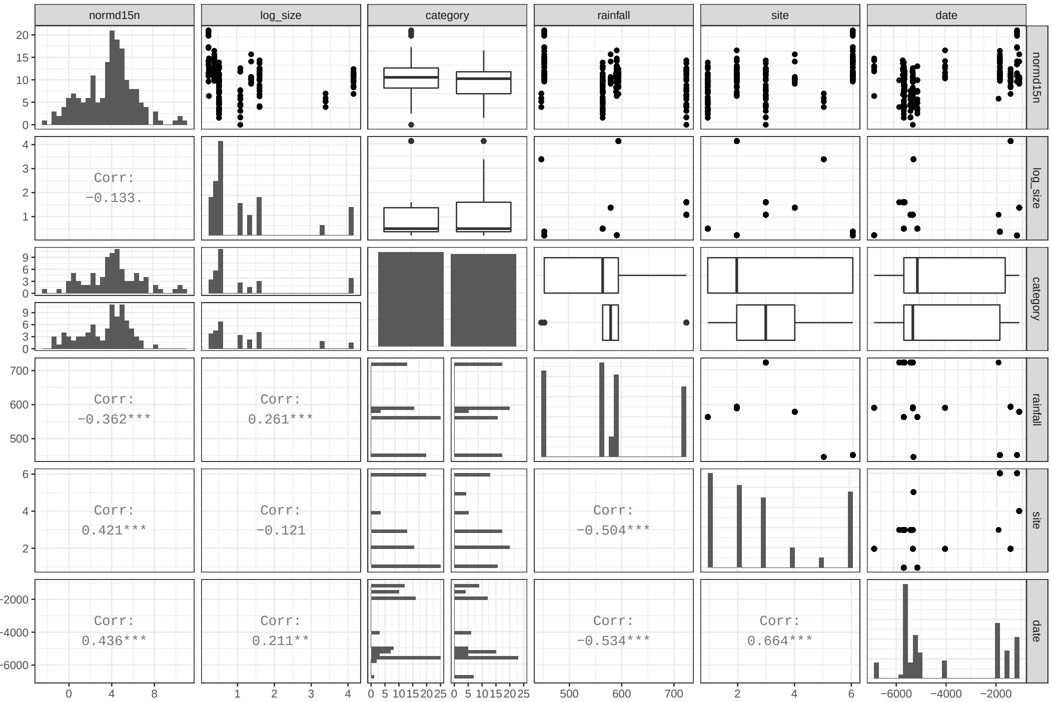

Figure 12: Pairwise relation between key variables in the archaeological data from the Greece (`aegean`)

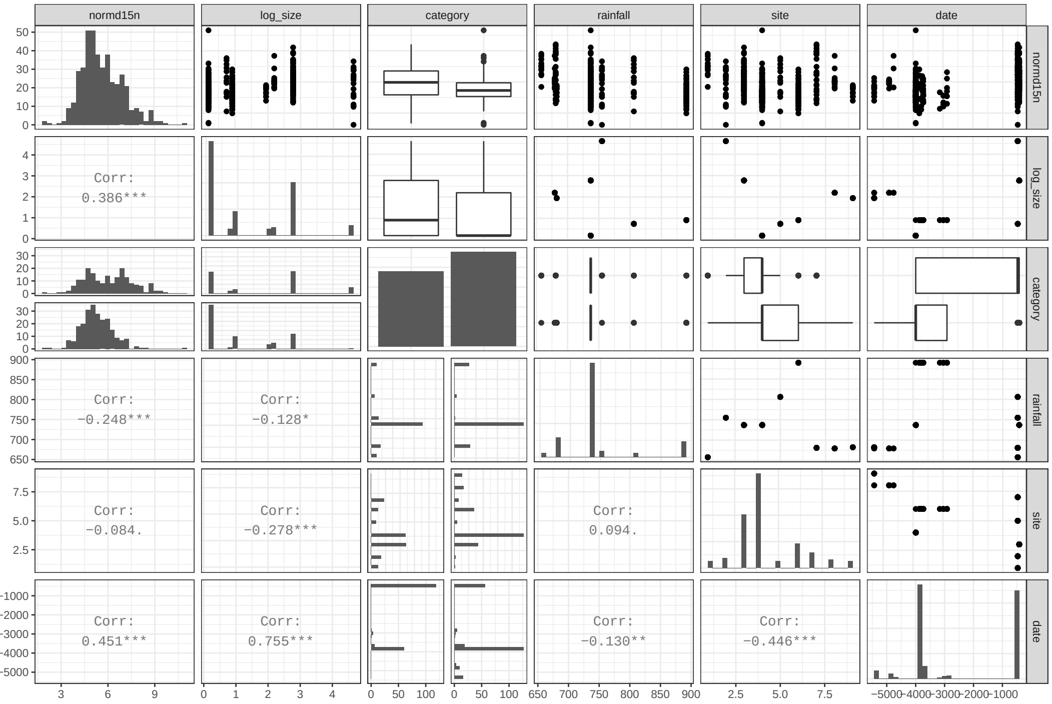

Figure 13: Pairwise relation between key variables in the archaeological data from the Germany (`swgermany`)

Appendix B. MCMC convergence analysis for SMI

Here we show simple convergence plots for the MCMC of the SMI posterior under the best η. The MCMC was ran for a total of 310,000 iterations, with a warm-up period of 10,000 and a thinning of 10. fig. 14 show the traces for the main parameters in the model, showing no sign of trending. Parameter correlations are displayed as a heatmap in fig. 15.

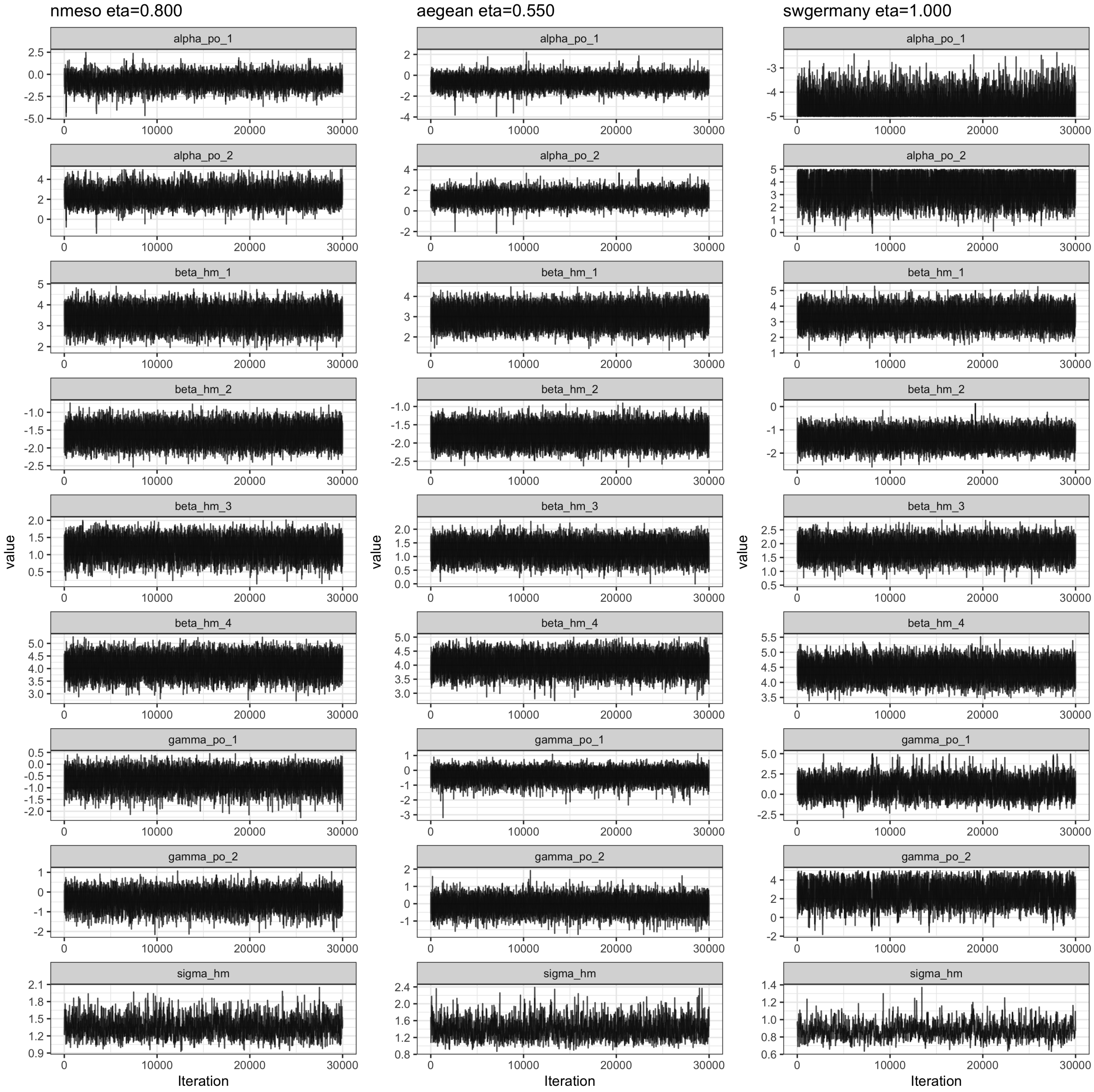

Figure 14: Trace plot of the Monte Carlo samples for the main parameters in the model.

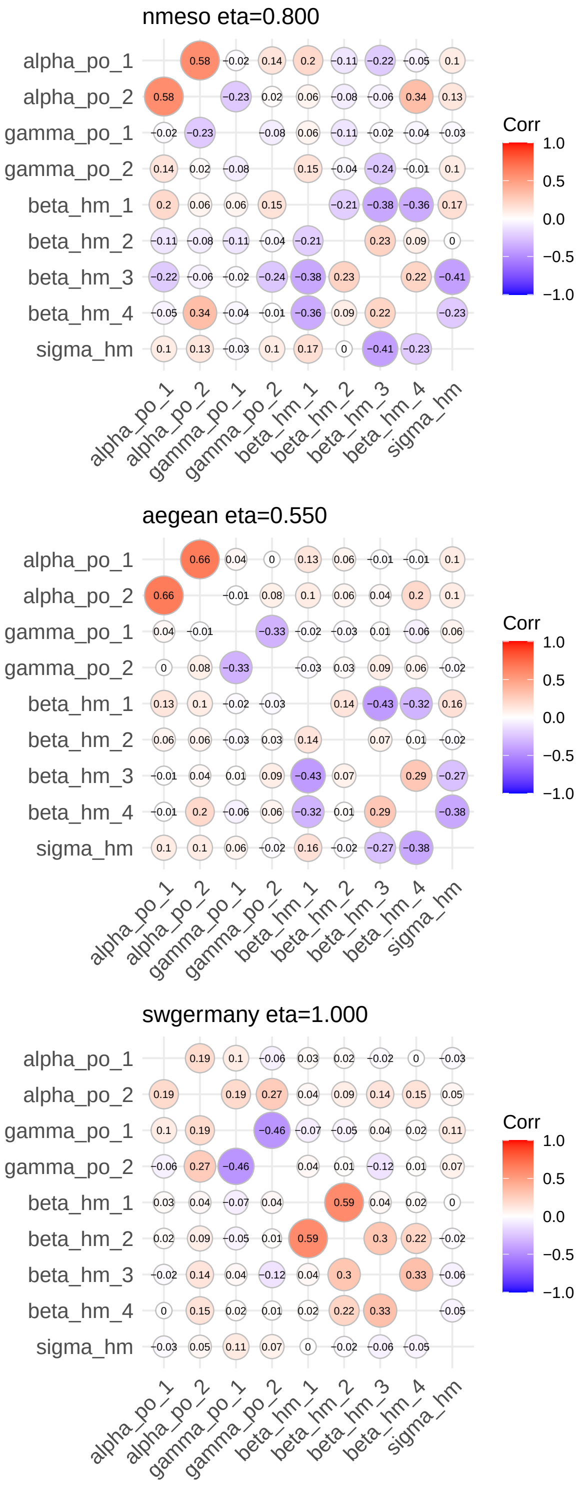

Figure 15: Heatmap with correlations of the principal parameters in the model (`gamma_po_1` corresponds to γ, the effect due to `size` in `PO`, whereas `gamma_po_2` is the effect due to `date`, denoted by τ in the text.)
